# Novel 4-Acrylamido-Quinoline Derivatives as Potent PI3K/mTOR Dual Inhibitors: The Design, Synthesis, and *in vitro* and *in vivo* Biological Evaluation

**DOI:** 10.3389/fchem.2019.00236

**Published:** 2019-04-24

**Authors:** Xiaodong Ma, Li Shen, Jiankang Zhang, Guoqiang Liu, Shuyu Zhan, Baoyue Ding, Xiaoqing Lv

**Affiliations:** ^1^School of Pharmacy, Anhui University of Chinese Medicine, Hefei, China; ^2^Department of Medicinal Chemistry, Anhui Academy of Chinese Medicine, Hefei, China; ^3^Ocean College, Zhejiang University, Zhoushan, China; ^4^Zhejiang University City College, Hangzhou, China; ^5^College of Medicine, Jiaxing University, Jiaxing, China

**Keywords:** 4-acrylamido-quinolines, PI3K/mTOR dual inhibitors, anti-proliferative activity, PI3K/Akt/mTOR signaling, metabolic stability, PK study

## Abstract

A novel structural series of quinoline derivatives were designed, synthesized and biologically evaluated as PI3K/mTOR dual inhibitors upon incorporation of C-4 acrylamide fragment. Consequently, all of them exerted remarkable inhibition against PI3Kα with IC_50_ values ranging from 0.50 to 2.03 nM. Besides, they exhibited sub-micromolar to low micromolar anti-proliferative activity against both prostate cancer PC3 and colorectal cancer HCT116 cell lines. In subsequent profiling, **8i**, a representative compound throughout this series, also significantly inhibited other class I PI3Ks and mTOR. In PC3 cells, it remarkably down-regulated the crucial biomarkers of PI3K/Akt/mTOR signaling, including phos-Akt (Ser473), phos-Akt (Thr308), phos-S6 ribosomal protein (Ser235/236), and phos-4E-BP1 (Thr37/46), at a concentration as low as 5 nM. Moreover, **8i** displayed favorable metabolic stability with long elimination half-life in both human liver and rat liver microsomes. A further *in vivo* pharmacokinetic (PK) study demonstrated **8i** possessed acceptable oral exposure, peak plasma concentration, and elimination half-life. Taken together, **8i**, as a potent PI3K/mTOR dual inhibitor, merited further investigation and structural optimization.

## Introduction

Aberrant activation of the phosphoinositide 3-kinase/Akt/mammalian target of rapamycin (PI3K/Akt/mTOR, PAM) signaling is regarded as a crucial hallmark in a broad spectrum of human cancers (Folkes et al., 2008; Furet et al., 2013). Among the kinases relevant to the cascade, PI3Kα, a member of class I PI3K subfamily, has spurred significant pharmaceutical investment in exploring its inactivating agents, since the identification of oncogenic *PIK3CA*, the gene encoding its catalytic subunit (Ma and Hu, 2013; Ndubaku et al., 2013; Heffron et al., 2016; Yadav et al., 2016). Owing to the high sequence homology shared by the four class I PI3K members within the ATP-binding pocket, numerous clinically developed PI3Kα modulators also concurrently inhibit PI3Kβ, PI3Kγ, and PI3Kδ, thereby termed as pan-class I PI3K inhibitors. As a signaling effector located downstream of PI3K, mTOR is also intimately associated with cancer initiation and development (Yang et al., 2013; Pike et al., 2015). There is abundant evidence demonstrating that mTOR is relevant to multiple negative feedback loops, especially mTOR-S6K1-PI3K signaling (Liu et al., 2011; Ma et al., 2015). Consequently, the sole inhibition of mTOR may activate PI3K through S6K1 and IRS-1, thus restarting cancer development and mediating drug resistance (Liu et al., 2010). Meanwhile, mTOR inhibition has been testified to be beneficial for the sensitivity to PI3K inhibitors (Elkabets et al., 2013). In view of the evidence implicating both PI3K and mTOR in cancer, as well as the potential to obtain synergism and address drug resistance (Stauffer et al., 2008; Ma and Hu, 2013; Beaufils et al., 2017; Ma et al., 2018), simultaneous inhibition of PI3K and mTOR is expected to provide therapeutic advantages over mono-inhibition of PI3K or mTOR. So far, several PI3K/mTOR dual inhibitors have been advanced into clinical trials, as exemplified by GSK2126458 dactolisisb, bimiralisib, gedatolisib, apitolisib, voxtalisib, PF04691502, BGT226, GDC-0084, LY3023414, and VS-5584 (Garces and Stocks, 2019).

**Figure 1:**
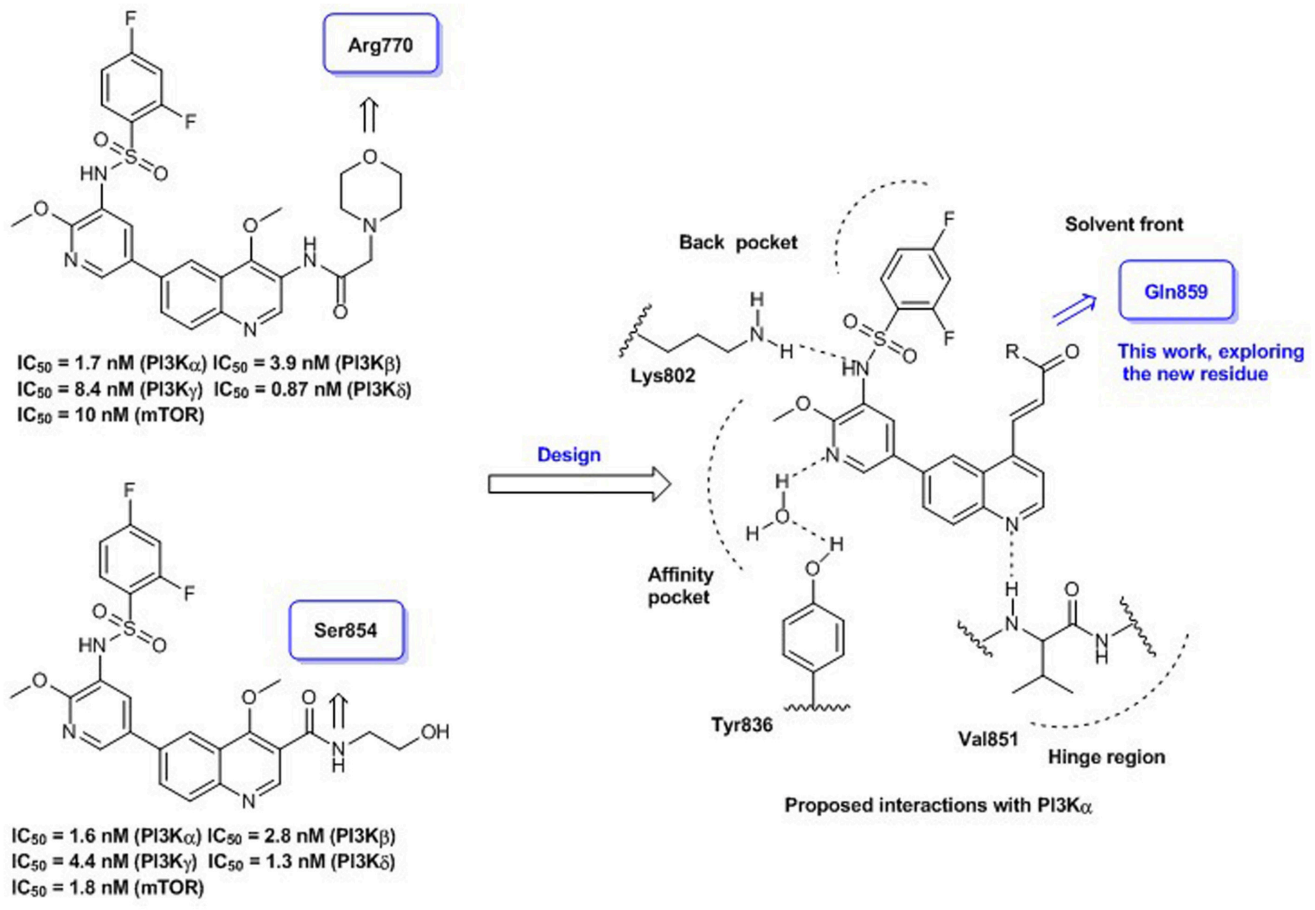
Quinoline-based PI3K/mTOR dual inhibitors obtained *via* probing residues at the entrance to PI3Kα active site: our previous and current work.

Quinoline represents a well-established template for constructing PI3K/mTOR dual inhibitors, and our efforts in search for PI3K signaling modulators belonging to this class have culminated in the discovery of numerous quinoline derivatives with attractive biological profiles (Zhang et al., 2017a,b). Although C-3 and C-4 positions of the quinoline template are oriented toward the entrance to PI3Kα active site, some surrounding residues can be exploited for producing additional interaction and enhancing the binding affinity. Previously, we have discovered some promising quinolines *via* exploring H-bond contact with residue Arg770 or Ser854 at this region upon structural elaboration at the C-3 position (Figure 1). To further broaden the chemical diversity of the quinoline-based PI3K/mTOR dual inhibitors, our recent medicinal chemistry efforts prioritize introduction of various acrylamide functionalities as the C-4 replacements for probing residue Gln859 at the entrance to the PI3Kα active site. The rationale for introducing the C-4 acrylamide functionality was based on the molecular docking analysis, which indicated its potential to confer H-bond interaction with residue Gln859. Moreover, a wide variety of terminal moieties of the C-4 acrylamide fragment were investigated for adjusting physicochemical properties. Hence, we herein communicate our work that has led to the discovery of a novel series of 4-acrylamido-quinoline derivatives as potent PI3K/mTOR dual inhibitors.

## Materials and Methods

### Chemistry

In this research, chemical reagents were commercially available, and, if necessary, pretreatment was carried out. With tetramethylsilane as the internal standard, ^1^H NMR and ^13^C NMR spectra were recorded on the 500 and 400 MHz instrument (Bruker Bioscience, Billerica, MA, USA), respectively. Chemical shifts (δ) were given in ppm and coupling constants (J) provided in hertz (Hz). ESI-MS data were measured on an Esquire-LC-00075 spectrometer, while HRMS data were collected by Waters Q-TOF Micromass. Column chromatography for the purification of intermediates or target compounds was performed using silica gel (200–300 mesh).

#### 6-Bromo-4-Methylquinoline (2)

4-Bromoaniline (33.0 g, 193.02 mmol) was added to a three-neck round bottom flask with acetic acid (200 mL). After FeCl_3_ (32.0 g, 198.96 mmol) was added, the mixture was stirred at room temperature for 10 min. Subsequently, methyl vinyl ketone (17.0 mL, 209.71 mmol) was added dropwise over 30 min and the reaction maintained at 70°C for 3 h. Then, ZnCl_2_ (26.0 g, 194.22 mmol) was added and the mixture refluxed for 2 h. After cooling to room temperature, the mixture was evaporated under reduced pressure, basified with 1N NaOH solution, and extracted with EA. The combined organic extracts were dried over magnesium sulfate and concentrated to give the crude product, which was further purified by column chromatography (EA/PE = 1:5) to afford the title intermediate (6.78 g, 30.68 mmol; yield 16%) as a brown solid. ^1^H NMR (500 MHz, DMSO-*d*_6_): δ 8.79 (d, *J* = 4.5 Hz, 1H, Ar-H), 8.29 (d, *J* = 2.0 Hz, 1H, Ar-H), 7.96 (d, *J* = 9.0 Hz, 1H, Ar-H), 7.88 (dd, *J* = 9.0, 2.0 Hz, 1H, Ar-H), 7.43 (d, *J* = 4.5 Hz, 1H, Ar-H), 2.67 (s, 3H, CH_3_). ESI-MS: m/z = 222 [M+H]^+^.

#### 6-Bromoquinoline-4-Carbaldehyde (3)

SeO_2_ (2.5 g, 22.34 mmol) was added to a solution of 6-bromo-4-methylquinoline (1.0 g, 4.52 mmol) in the mixture of dioxane/H_2_O (8/1, V/V) at room temperature. After being stirred at 100°C for 2 h, the reaction mixture was filtered and the filtrate was concentrated under reduced pressure. The residue was dissolved in EA and washed successively with saturated aqueous NaHCO_3_ and water. The organic phase was then dried with magnesium sulfate and concentrated in vacuo to afford a brown solid, which was purified by column chromatography (EA/PE = 1:5) to give 6-bromoquinoline-4-carbaldehyde (0.78 g, 3.32 mmol; yield 73%) as a light yellow solid. ^1^H NMR (500 MHz, DMSO-*d*_6_): δ 10.49 (s, 1H, CHO), 9.28 (d, *J* = 4.5 Hz, 1H, Ar-H), 9.18 (d, *J* = 2.0 Hz, 1H, Ar-H), 8.12 (d, *J* = 9.0 Hz, 1H, Ar-H), 8.11 (d, *J* = 4.5 Hz, 1H, Ar-H), 8.03 (dd, *J* = 9.0, 2.0 Hz, 1H, Ar-H). ESI-MS: m/z = 236 [M+H]^+^.

#### Ethyl (*E*)-3-(6-Bromoquinolin-4-yl)acrylate (4)

Triethyl phosphonoacetate (350 mg, 1.56 mmol) was added to a suspension of NaH (100 mg, 60%, 2.50 mmol) in THF at 0°C, and the resultant mixture was stirred for 30 min. 6-bromoquinoline-4-carbaldehyde (300 mg, 1.28 mmol) was then added and the mixture stirred for 1 h at room temperature. The reaction mixture was cooled to 0°C, and ice water was added. After extracting with EA, the organic phase was washed with saturated aqueous NaHCO_3_, dried with magnesium sulfate and concentrated in vacuo to afford the crude product, which was further purified by column chromatography (EA/PE = 1:5) to give the title intermediate (342 mg, 1.12 mmol; yield 88%) as a white solid. ^1^H NMR (500 MHz, DMSO-*d*_6_): δ 8.98 (d, *J* = 4.5 Hz, 1H, Ar-H), 8.48 (d, *J* = 2.0 Hz, 1H, Ar-H), 8.36 (d, *J* = 16.0 Hz, 1H, alkene hydrogen), 8.03 (d, *J* = 9.0 Hz, 1H, Ar-H), 7.97–7.95 (dd, *J* = 9.0, 2.0 Hz, 1H, Ar-H), 7.93 (d, *J* = 4.5 Hz, 1H, Ar-H), 6.90 (d, *J* = 16.0 Hz, 1H, alkene hydrogen), 4.28 (q, *J* = 7.0 Hz, 2H, OCH_2_), 1.32 (t, *J* = 7.0 Hz, 3H, CH_3_). ESI-MS: m/z = 306 [M+H]^+^.

#### (*E*)-3-(6-Bromoquinolin-4-yl)acrylic Acid (5)

Ethyl (*E*)-3-(6-bromoquinolin-4-yl)acrylate (306 mg, 1.00 mmol) and 2.5 N NaOH (20 mL) were charged in a round bottom flask. The mixture was stirred under reflux for 2 h. After cooling to room temperature, the pH of the mixture was adjusted to 5 with 2 N HCl, and the resultant solid was filtered and washed with water. The filter cake was then dried under reduced pressure to afford the title intermediate (241 mg, 0.87 mmol; yield 87%) as a white solid. ^1^H NMR (500 MHz, DMSO-*d*_6_): δ 12.89 (s, 1H, COOH), 8.97 (d, *J* = 4.5 Hz, 1H, Ar-H), 8.45 (d, *J* = 2.0 Hz, 1H, Ar-H), 8.29 (d, *J* = 16.0 Hz, 1H, alkene hydrogen), 8.02 (d, *J* = 9.0 Hz, 1H, Ar-H), 7.95 (dd, *J* = 9.0, 2.0 Hz, 1H, Ar-H), 7.90 (d, *J* = 4.5 Hz, 1H, Ar-H), 6.80 (d, *J* = 16.0 Hz, 1H, alkene hydrogen). ESI-MS: m/z = 278 [M+H]^+^.

#### General Synthetic Procedure A for the Intermediates 6a-o

A solution of (*E*)-3-(6-bromoquinolin-4-yl)acrylic acid (1.0 equiv), EDCI (1.5 equiv) and HOBt (1.0 equiv) in dry DCM was stirred at room temperature for 2 h. TEA (3.0 equiv) and corresponding amine (2.0 equiv) were then added, and the resultant mixture was stirred for 1 h. After this, it was successively washed with 1N NaOH and water. The organic phase was dried with magnesium sulfate and concentrated in vacuo to afford the crude product, which was further purified by column chromatography to give the desired compounds.

##### (E)-3-(6-bromoquinolin-4-yl)-N-(tert-butyl)acrylamide(6a)

The title intermediate was prepared from **5** (100 mg, 0.36 mmol) and *tert*-butylamine (53 mg, 0.72 mmol) according to the general synthetic procedure A, as a white solid (98 mg, 0.30 mmol; yield 83%). ^1^H NMR (500 MHz, DMSO-*d*_6_): δ 8.96 (d, *J* = 4.5 Hz, 1H, Ar-H), 8.40 (brs, 1H, NH), 8.10–7.98 (m, 3H, Ar-H × 2 + alkene hydrogen), 7.95 (dd, *J* = 9.0, 2.0 Hz, 1H, Ar-H), 7.68 (d, *J* = 4.5 Hz, 1H, Ar-H), 6.91 (d, *J* = 15.5 Hz, 1H, alkene hydrogen), 1.37 (s, 9H, CH_3_ × 3). ESI-MS: m/z = 333 [M+H]^+^.

##### (E)-3-(6-bromoquinolin-4-yl)-N-(2-methoxyethyl)acrylamide(6b)

The title intermediate was prepared from **5** (100 mg, 0.36 mmol) and 2-methoxyethan-1-amine (54 mg, 0.72 mmol) according to the general synthetic procedure A, as a white solid (76 mg, 0.23 mmol; yield 64%). ^1^H NMR (500 MHz, DMSO-*d*_6_): δ 8.96 (d, *J* = 4.5 Hz, 1H, Ar-H), 8.51 (brs, 1H, NH), 8.43 (brs, 1H, Ar-H), 8.09 (d, *J* = 15.5 Hz, 1H, alkene hydrogen), 8.02 (d, *J* = 9.0 Hz, 1H, Ar-H), 7.94 (brd, *J* = 9.0 Hz, 1H, Ar-H), 7.72 (d, *J* = 4.5 Hz, 1H, Ar-H), 6.91 (d, *J* = 15.5 Hz, 1H, alkene hydrogen), 3.48–3.40 (m, 4H, CH_2_ × 2), 3.30 (s, 3H, OCH_3_). ESI-MS: m/z = 335 [M+H]^+^.

##### (E)-3-(6-bromoquinolin-4-yl)-N-(2-hydroxyethyl)-N-methylacrylamide(6c)

The title intermediate was prepared from **5** (100 mg, 0.36 mmol) and 2-(methylamino)ethan-1-ol (54 mg, 0.72 mmol) according to the general synthetic procedure A, as a white solid (88 mg, 0.26 mmol; yield 72%). ^1^H NMR (500 MHz, DMSO-*d*_6_): δ 8.97 (d, *J* = 4.5 Hz, 1H, Ar-H), 8.40 (d, *J* = 2.0 Hz, 0.36H, Ar-H), 8.39 (d, *J* = 2.0 Hz, 0.65H, Ar-H), 8.14 (d, *J* = 15.5 Hz, 0.66H, alkene hydrogen), 8.10 (d, *J* = 15.5 Hz, 0.38H, alkene hydrogen), 8.02 (d, *J* = 9.0 Hz, 1H, Ar-H), 7.97–7.87 (m, 2H, Ar-H), 7.46 (d, *J* = 15.5 Hz, 0.63H, alkene hydrogen), 7.43 (d, *J* = 15.5 Hz, 0.38H, alkene hydrogen), 4.88 (t, *J* = 5.5 Hz, 0.68H, OH), 4.76 (t, *J* = 5.5 Hz, 0.37H, OH), 3.63 – 3.56 (m, 3.28H, CH_2_), 3.51 (t, *J* = 5.5 Hz, 0.70H, CH_2_), 3.24 (s, 1H, CH_3_), 3.01 (s, 2H, CH_3_). ESI-MS: m/z = 335 [M+H]^+^.

##### (E)-3-(6-bromoquinolin-4-yl)-N-cyclopropylacrylamide(6d)

The title intermediate was prepared from **5** (100 mg, 0.36 mmol) and cyclopropanamine (41 mg, 0.72 mmol) according to the general synthetic procedure A, as a white solid (93 mg, 0.29 mmol; yield 81%). ^1^H NMR (500 MHz, CDCl_3_): δ 8.95 (d, *J* = 4.5 Hz, 1H, Ar-H), 8.50 (brs, 1H, NH), 8.42 (d, *J* = 2.0 Hz, 1H, Ar-H), 8.07 (d, *J* = 15.5 Hz, 1H, alkene hydrogen), 8.01 (d, *J* = 9.0 Hz, 1H, Ar-H), 7.97–7.91 (dd, *J* = 9.0, 2.0 Hz, 1H, Ar-H), 7.71 (d, *J* = 4.5 Hz, 1H, Ar-H), 6.77 (d, *J* = 15.5 Hz, 1H, alkene hydrogen), 2.82 (m, 1H, CH), 0.73 (m, 2H, CH_2_), 0.53 (m, 2H, CH_2_). ESI-MS: m/z = 317 [M+H]^+^.

##### (E)-3-(6-bromoquinolin-4-yl)-N-cyclobutylacrylamide(6e)

The title intermediate was prepared from **5** (100 mg, 0.36 mmol) and cyclobutanamine (51 mg, 0.72 mmol) according to the general synthetic procedure A, as a white solid (102 mg, 0.31 mmol; yield 86%). ^1^H NMR (500 MHz, DMSO-*d*_6_): δ 8.96 (d, *J* = 4.5 Hz, 1H, Ar-H), 8.64 (d, *J* = 7.0 Hz, 1H, NH), 8.41 (brs, 1H, Ar-H), 8.07 (d, *J* = 15.5 Hz, 1H, alkene hydrogen), 8.02 (d, *J* = 9.0 Hz, 1H, Ar-H), 7.94 (brd, *J* = 9.0 Hz, 1H, Ar-H), 7.71 (d, *J* = 4.5 Hz, 1H, Ar-H), 6.80 (d, *J* = 15.5 Hz, 1H, alkene hydrogen), 4.35 (m, 1H, CH), 2.31–2.18 (m, 2H, CH_2_), 2.03–1.92 (m, 2H, CH_2_), 1.77–1.63 (m, 2H, CH_2_). ESI-MS: m/z = 331 [M+H]^+^.

##### (E)-3-(6-bromoquinolin-4-yl)-1-(pyrrolidin-1-yl)prop-2-en-1-one(6f)

The title intermediate was prepared from **5** (100 mg, 0.36 mmol) and pyrrolidine (51 mg, 0.72 mmol) according to the general synthetic procedure A, as a white solid (95 mg, 0.29 mmol; yield 81%). ^1^H NMR (500 MHz, DMSO-*d*_6_): δ 8.98 (d, *J* = 4.5 Hz, 1H, Ar-H), 8.40 (d, *J* = 2.0 Hz, 1H, Ar-H), 8.15 (d, *J* = 15.5 Hz, 1H, alkene hydrogen), 8.02 (d, *J* = 9.0 Hz, 1H, Ar-H), 7.94 (m, 2H, Ar-H), 7.26 (d, *J* = 15.5 Hz, 1H, alkene hydrogen), 3.70 (d, *J* = 6.5 Hz, 2H, CH_2_), 3.46 (d, *J* = 6.5 Hz, 2H, CH_2_), 1.95 (m, 2H, CH_2_), 1.85 (m, 2H, CH_2_). ESI-MS: m/z = 331 [M+H]^+^.

##### (E)-3-(6-bromoquinolin-4-yl)-1-(piperidin-1-yl)prop-2-en-1-one(6g)

The title intermediate was prepared from **5** (100 mg, 0.36 mmol) and piperidine (61 mg, 0.72 mmol) according to the general synthetic procedure A, as a white solid (113 mg, 0.33 mmol; yield 92%). ^1^H NMR (500 MHz, DMSO-*d*_6_): δ 8.96 (d, *J* = 4.5 Hz, 1H, Ar-H), 8.40 (d, *J* = 2.0 Hz, 1H, Ar-H), 8.13 (d, *J* = 15.5 Hz, 1H, alkene hydrogen), 8.01 (d, *J* = 9.0 Hz, 1H, Ar-H), 7.97 (d, *J* = 4.5 Hz, 1H, Ar-H), 7.93 (dd, *J* = 9.0, 2.0 Hz, 1H, Ar-H), 7.53 (d, *J* = 15.5 Hz, 1H, alkene hydrogen), 3.72–3.65 (m, 2H, CH_2_), 3.62–3.56 (m, 2H, CH_2_), 1.64 (m, 2H, CH_2_), 1.54 (m, 4H, CH_2_ × 2). ESI-MS: m/z = 345 [M+H]^+^.

##### (E)-3-(6-bromoquinolin-4-yl)-1-morpholinoprop-2-en-1-one(6h)

The title intermediate was prepared from **5** (100 mg, 0.36 mmol) and morpholine (63 mg, 0.72 mmol) according to the general synthetic procedure A, as a white solid (79 mg, 0.23 mmol; yield 64%). ^1^H NMR (500 MHz, DMSO-*d*_6_): δ 8.96 (d, *J* = 4.5 Hz, 1H, Ar-H), 8.41 (d, *J* = 2.0 Hz, 1H, Ar-H), 8.17 (d, *J* = 15.0 Hz, 1H, alkene hydrogen), 8.00 (d, *J* = 9.0 Hz, 1H, Ar-H), 7.96 (d, *J* = 4.5 Hz, 1H, Ar-H), 7.95–7.90 (dd, *J* = 9.0, 2.0 Hz, 1H, Ar-H), 7.51 (d, *J* = 15.0 Hz, 1H, alkene hydrogen), 3.74 (m, 2H, CH_2_), 3.62 (m, 6H, CH_2_ × 3). ESI-MS: m/z = 347 [M+H]^+^.

##### (E)-3-(6-bromoquinolin-4-yl)-1-(4-methylpiperazin-1-yl)prop-2-en-1-one(6i)

The title intermediate was prepared from **5** (100 mg, 0.36 mmol) and *N*-methylpiperazine (72 mg, 0.72 mmol) according to the general synthetic procedure A, as a white solid (81 mg, 0.23 mmol; yield 64%). ^1^H NMR (500 MHz, DMSO-*d*_6_): δ 8.96 (d, *J* = 4.5 Hz, 1H, Ar-H), 8.39 (d, *J* = 2.0 Hz, 1H, Ar-H), 8.15 (d, *J* = 15.5 Hz, 1H, alkene hydrogen), 8.00 (d, *J* = 9.0 Hz, 1H, Ar-H), 7.96 (d, *J* = 4.5 Hz, 1H, Ar-H), 7.92 (dd, *J* = 9.0, 2.0 Hz, 1H, Ar-H), 7.53 (d, *J* = 15.5 Hz, 1H, alkene hydrogen), 3.77–3.69 (m, 2H, CH_2_), 3.65–3.58 (m, 2H, CH_2_), 2.40–2.30 (m, 4H, CH_2_ × 2), 2.21 (s, 3H, CH_3_). ESI-MS: m/z = 360 [M+H]^+^.

##### (E)-3-(6-bromoquinolin-4-yl)-1-(4-isopropylpiperazin-1-yl)prop-2-en-1-one(6j)

The title intermediate was prepared from **5** (100 mg, 0.36 mmol) and 1-isopropylpiperazine (92 mg, 0.72 mmol) according to the general synthetic procedure A, as a white solid (61 mg, 0.16 mmol; yield 44%). ^1^H NMR (500 MHz, DMSO-*d*_6_): δ 8.97 (d, *J* = 4.5 Hz, 1H, Ar-H), 8.41 (brs, 1H, Ar-H), 8.15 (d, *J* = 15.5 Hz, 1H, alkene hydrogen), 8.01 (d, *J* = 9.0 Hz, 1H, Ar-H), 7.97 (d, *J* = 4.5 Hz, 1H, Ar-H), 7.91 (dd, *J* = 9.0, 2.0 Hz, 1H, Ar-H), 7.53 (d, *J* = 15.5 Hz, 1H, alkene hydrogen), 3.70 (m, 2H, CH_2_), 3.60 (m, 2H, CH_2_), 2.70 (m, 1H, CH), 2.47 (m, 4H, CH_2_ × 2), 0.99 (s, 3H, CH_3_), 0.98 (s, 3H, CH_3_). ESI-MS: m/z = 388 [M+H]^+^.

##### (E)-3-(6-bromoquinolin-4-yl)-1-(4-hydroxypiperidin-1-yl)prop-2-en-1-one (6k)

The title intermediate was prepared from **5** (100 mg, 0.36 mmol) and 4-hydroxypiperidine (73 mg, 0.72 mmol) according to the general synthetic procedure A, as a white solid (88 mg, 0.24 mmol; yield 67%). ^1^H NMR (500 MHz, DMSO-*d*_6_): δ 8.94 (d, *J* = 4.5 Hz, 1H, Ar-H), 8.38 (d, *J* = 2.0 Hz, 1H, Ar-H), 8.11 (d, *J* = 15.5 Hz, 1H, alkene hydrogen), 7.99 (d, *J* = 9.0 Hz, 1H, Ar-H), 7.95 (d, *J* = 4.5 Hz, 1H, Ar-H), 7.91 (dd, *J* = 9.0, 2.0 Hz, 1H, Ar-H), 7.52 (d, *J* = 15.5 Hz, 1H, alkene hydrogen), 4.79 (d, *J* = 4.0 Hz, 1H, OH), 4.08–3.94 (m, 2H, CH_2_), 3.75 (m, 1H, CH_2_), 3.44–3.36 (m, 1H, CH_2_), 3.26–3.13 (m, 1H, CH_2_), 1.78 (m, 2H, CH_2_), 1.47–1.28 (m, 2H, CH_2_). ESI-MS: m/z = 361 [M+H]^+^.

##### (E)-3-(6-bromoquinolin-4-yl)-1-(3-hydroxypiperidin-1-yl)prop-2-en-1-one(6l)

The title intermediate was prepared from **5** (100 mg, 0.36 mmol) and 3-hydroxypiperidine (73 mg, 0.72 mmol) according to the general synthetic procedure A, as a white solid (96 mg, 0.27 mmol; yield 75%). ^1^H NMR (500 MHz, DMSO-*d*_6_): δ 8.97 (d, *J* = 4.0 Hz, 1H, Ar-H), 8.41 (brs, 1H, Ar-H), 8.11 (dd, *J* = 15.5, 6.0 Hz, 1H, alkene hydrogen), 7.98 (m, 3H, Ar-H), 7.51 (t, *J* = 15.5 Hz, 1H, alkene hydrogen), 5.01 (d, *J* = 3.5 Hz, 0.48H, OH), 4.88 (d, *J* = 3.5 Hz, 0.57H, OH), 4.27 (m, 0.49H, CH), 3.99 (m, 0.49H, CH), 3.80 (m, 0.56H, CH_2_), 3.63 (m, 1H, CH_2_), 3.54–3.40 (m, 1.41H, CH_2_), 3.27–3.12 (m, 0.63H, CH_2_), 2.86–2.70 (m, 0.50H, CH_2_), 1.75 (m, 2H, CH_2_), 1.41 (m, 2H, CH_2_). ESI-MS: m/z = 361 [M+H]^+^.

##### (E)-3-(6-bromoquinolin-4-yl)-1-(4-(hydroxymethyl)piperidin-1-yl)prop-2-en-1-one(6m)

The title intermediate was prepared from **5** (100 mg, 0.36 mmol) and piperidin-4-ylmethanol (83 mg, 0.72 mmol) according to the general synthetic procedure A, as a white solid (72 mg, 0.19 mmol; yield 53%). ^1^H NMR (500 MHz, DMSO-*d*_6_): δ 8.96 (brs, 1H, Ar-H), 8.41 (brs, 1H, Ar-H), 8.13 (d, *J* = 15.5 Hz, 1H, alkene hydrogen), 8.06–7.88 (m, 3H, Ar-H), 7.54 (d, *J* = 15.5 Hz, 1H, alkene hydrogen), 4.52 (m, 2H, CH_2_), 4.28 (d, *J* = 12.5 Hz, 1H, OH), 3.28 (m, 2H, CH_2_), 3.10 (m, 1H, CH_2_), 2.70 (m, 1H, CH_2_), 1.80–1.61 (m, 3H, CH + CH_2_), 1.16–1.01 (m, 2H, CH_2_). ESI-MS: m/z = 375 [M+H]^+^.

##### (E)-3-(6-bromoquinolin-4-yl)-N-(2-morpholinoethyl)acrylamide(6n)

The title intermediate was prepared from **5** (100 mg, 0.36 mmol) and 2-morpholinoethan-1-amine (94 mg, 0.72 mmol) according to the general synthetic procedure A, as a white solid (85 mg, 0.22 mmol; yield 61%). ^1^H NMR (500 MHz, DMSO-*d*_6_): δ 8.96 (d, *J* = 4.5 Hz, 1H, Ar-H), 8.43 (d, *J* = 2.0 Hz, 1H, Ar-H), 8.37 (d, *J* = 5.0 Hz, 1H, NH), 8.08 (d, *J* = 15.5 Hz, 1H, alkene hydrogen), 8.02 (d, *J* = 9.0 Hz, 1H, Ar-H), 7.96 (dd, *J* = 9.0, 2.0 Hz, 1H, Ar-H), 7.74 (d, *J* = 4.5 Hz, 1H, Ar-H), 6.90 (d, *J* = 15.5 Hz, 1H, alkene hydrogen), 3.63–3.55 (m, 4H, CH_2_ × 2), 3.37 (s, 2H, CH_2_), 2.48–2.34 (m, 6H, CH_2_ × 3). ESI-MS: m/z = 390 [M+H]^+^.

##### (E)-3-(6-bromoquinolin-4-yl)-N-phenylacrylamide(6o)

The title intermediate was prepared from **5** (100 mg, 0.36 mmol) and aniline (67 mg, 0.72 mmol) according to the general synthetic procedure A, as a white solid (68 mg, 0.19 mmol; yield 53%). ^1^H NMR (500 MHz, DMSO-*d*_6_): δ 10.45 (s, 1H, NH), 9.00 (d, *J* = 4.5 Hz, 1H, Ar-H), 8.49 (d, *J* = 2.0 Hz, 1H, Ar-H), 8.26 (d, *J* = 15.5 Hz, 1H, alkene hydrogen), 8.04 (d, *J* = 9.0 Hz, 1H, Ar-H), 7.99–7.93 (dd, *J* = 9.0, 2.0 Hz, 1H, Ar-H), 7.79 (d, *J* = 4.5 Hz, 1H, Ar-H), 7.75 (d, *J* = 8.0 Hz, 2H, Ar-H), 7.38 (t, *J* = 8.0 Hz, 2H, Ar-H), 7.13 (m, 1H, Ar-H), 7.07 (d, *J* = 15.5 Hz, 1H, alkene hydrogen). ESI-MS: m/z = 353 [M+H]^+^.

#### General Synthetic Procedure B for the Target Compounds 8a-o and 10

To a three-neck round bottom flask was added the aryl bromide (1.0 equiv), 2,4-difluoro-*N*-(2-methoxy-5-(4,4,5,5-tetramethyl-1,3,2-dioxaborolan-2-yl)pyridin-3-yl)benzenesulfonamide **7** (1.0 equiv), Pd(dppf)_2_Cl_2_ (0.1 equiv), K_2_CO_3_ (3.0 equiv) and dioxane/H_2_O (3/1, V/V). The flask was fitted with a N_2_ inlet adaptor and purged with N_2_ for 15 min. The reaction mixture was then sealed under N_2_ atmosphere and stirred at 100°C for 10 h. The mixture was concentrated under reduced pressure, and the residue was dissolved in DCM, washed with water twice and dried over magnesium sulfate. The crude product was purified by column chromatography to yield the desired target compound. The copies of NMR and HRMS spectra are provided in the Supplementary Material.

##### (E)-N-(tert-butyl)-3-(6-(5-((2,4-difluorophenyl)sulfonamido)-6-methoxypyridin-3-yl)quinolin-4-yl)acrylamide(8a)

The title compound was prepared from **6a** (53 mg, 0.16 mmol) and **7** (68 mg, 0.16 mmol) according to the general synthetic procedure B, as an off-white solid (27 mg, 0.049 mmol; yield 31%). ^1^H NMR (500 MHz, DMSO-*d*_6_): δ 10.36 (s, 1H, NH), 8.94 (d, *J* = 4.5 Hz, 1H, Ar-H), 8.50 (brs, 1H, NH), 8.31 (d, *J* = 2.0 Hz, 1H, Ar-H), 8.24 (d, *J* = 15.5 Hz, 1H, alkene hydrogen), 8.15 (d, *J* = 9.0 Hz, 1H, Ar-H), 8.05 (m, 3H, Ar-H), 7.83 (m, 1H, Ar-H), 7.67 (d, *J* = 4.5 Hz, 1H, Ar-H), 7.62 (m, 1H, Ar-H), 7.26 (m, 1H, Ar-H), 6.96 (d, *J* = 15.5 Hz, 1H, alkene hydrogen), 3.70 (s, 3H, OCH_3_), 1.37 (s, 9H, CH_3_ × 3). ^13^C NMR (100 MHz, DMSO-*d*_6_): δ 165.41, 165.07 (dd, *J*_C−F_ = 254.4, 12.3 Hz), 159.34 (dd, *J*_C−F_ = 257.9, 12.9 Hz), 157.59, 150.51, 147.49, 142.78, 140.69, 134.87, 133.95, 132.81, 131.88 (d, *J*_C−F_ = 11.0 Hz), 130.49, 129.09, 128.94, 128.55, 125.83, 125.12 (dd, *J*_C−F_ = 16.1, 3.5 Hz), 120.92, 119.91, 118.64, 111.87 (dd, *J*_C−F_ = 22.0, 3.4 Hz), 105.80 (t, *J*_C−F_ = 26.1 Hz), 53.42, 22.62, 5.86. HRMS (ESI) m/z calcd for C_28_H_27_F_2_N_4_O_4_S [M + H]^+^ 553.1721, found 553.1725.

##### (E)-3-(6-(5-((2,4-difluorophenyl)sulfonamido)-6-methoxypyridin-3-yl)quinolin-4-yl)-N-(2-methoxyethyl)acrylamide(8b)

The title compound was prepared from **6b** (53 mg, 0.16 mmol) and **7** (68 mg, 0.16 mmol) according to the general synthetic procedure B, as an off-white solid (18 mg, 0.032 mmol; yield 20%). ^1^H NMR (500 MHz, DMSO-*d*_6_): δ 10.36 (s, 1H, NH), 8.94 (d, *J* = 4.5 Hz, 1H, Ar-H), 8.53 (brs, 1H, Ar-H), 8.50 (t, *J* = 5.5 Hz, 1H, NH), 8.35 (d, *J* = 1.5 Hz, 1H, Ar-H), 8.30 (d, *J* = 15.5 Hz, 1H, alkene hydrogen), 8.16 (d, *J* = 9.0 Hz, 1H, Ar-H), 8.10–8.04 (m, 2H, Ar-H), 7.82 (m, 1H, Ar-H), 7.72 (d, *J* = 4.5 Hz, 1H, Ar-H), 7.60 (m, 1H, Ar-H), 7.25 (td, *J* = 8.5, 2.5 Hz, 1H, Ar-H), 6.96 (d, *J* = 15.5 Hz, 1H, alkene hydrogen), 3.70 (s, 3H, OCH_3_), 3.45 (m, 4H, CH2 × 2), 3.30 (s, 3H, OCH_3_). ^13^C NMR (100 MHz, DMSO-*d*_6_): δ 165.08 (dd, *J*_C−F_ = 254.0, 11.7 Hz), 164.40, 159.34 (dd, *J*_C−F_ = 257.5, 13.6 Hz), 157.57, 150.57, 147.50, 142.87, 140.65, 134.86, 133.95, 133.04, 131.88 (d, *J*_C−F_ = 11.4 Hz), 130.50, 129.10, 129.06, 128.53, 125.82, 125.06 (dd, *J*_C−F_ = 14.8, 4.0 Hz), 120.91, 119.82, 118.66, 111.87 (dd, *J*_C−F_ = 25.6, 3.9 Hz), 105.80 (t, *J*_C−F_ = 26.4 Hz), 70.57, 57.89, 53.42, 38.72. HRMS (ESI) m/z calcd for C_27_H_25_F_2_N_4_O_5_S [M + H]^+^ 555.1513, found 555.1510.

##### (E)-3-(6-(5-((2,4-difluorophenyl)sulfonamido)-6-methoxypyridin-3-yl)quinolin-4-yl)-N-(2-hydroxyethyl)-N-methylacrylamide(8c)

The title compound was prepared from **6c** (53 mg, 0.16 mmol) and **7** (68 mg, 0.16 mmol) according to the general synthetic procedure B, as an off-white solid (20 mg, 0.036 mmol; yield 23%). ^1^H NMR (500 MHz, DMSO-*d*_6_): δ 8.90 (d, *J* = 4.5 Hz, 1H, Ar-H), 8.30 (d, *J* = 15.5 Hz, 0.44H, alkene hydrogen), 8.26 (d, *J* = 15.5 Hz, 0.56H, alkene hydrogen), 8.13 (brs, 1H, Ar-H), 8.10 (d, *J* = 8.5 Hz, 1H, Ar-H), 8.01–7.84 (m, 4H, Ar-H), 7.70 (brs, 1H, Ar-H), 7.52 (d, *J* = 15.5 Hz, 0.45H, alkene hydrogen), 7.49 (d, *J* = 15.5 Hz, 0.56H, alkene hydrogen), 7.29 (brs, 1H, Ar-H), 7.21 (m, 1H, Ar-H), 4.92 (brs, 0.62H, OH), 4.78 (t, *J* = 5.5 Hz, 0.44H, OH), 3.79 (s, 3H, OCH_3_), 3.63 (m, 3.22H, CH_2_), 3.53 (t, *J* = 6.0 Hz, 0.80H, CH_2_), 3.28 (s, 1H, CH_3_), 3.04 (s, 2H, CH_3_). HRMS (ESI) m/z calcd for C_27_H_25_F_2_N_4_O_5_S [M + H]^+^ 555.1513, found 555.1516.

##### (E)-N-cyclopropyl-3-(6-(5-((2,4-difluorophenyl)sulfonamido)-6-methoxypyridin-3-yl)quinolin-4-yl)acrylamide(8d)

The title compound was prepared from **6d** (50 mg, 0.16 mmol) and **7** (68 mg, 0.16 mmol) according to the general synthetic procedure B, as an off-white solid (18 mg, 0.034 mmol; yield 21%). ^1^H NMR (500 MHz, CDCl_3_): δ 8.84 (brs, 1H, NH), 8.27 (d, *J* = 15.0 Hz, 1H, alkene hydrogen), 8.18–8.08 (m, 3H, Ar-H), 7.99 (d, *J* = 2.0 Hz, 1H, Ar-H), 7.91 (m, 1H, Ar-H), 7.82 (d, *J* = 8.5 Hz, 1H, Ar-H), 7.45 (brs, 1H, Ar-H), 7.28 (brs, 1H, Ar-H), 7.07 (m, 1H, Ar-H), 6.90 (m, 1H, Ar-H), 6.54 (d, *J* = 15.0 Hz, 1H, alkene hydrogen), 5.99 (brs, 1H, NH), 3.91 (s, 3H, OCH_3_), 2.87 (m, 1H, CH), 0.82 (m, 2H, CH_2_), 0.58 (m, 2H, CH_2_). ^13^C NMR (100 MHz, DMSO-*d*_6_): δ 165.10 (dd, *J*_C−F_ = 253.7, 12.0 Hz), 163.56, 159.35 (dd, *J*_C−F_ = 257.6, 13.3 Hz), 157.51, 150.49, 147.53, 142.81, 140.84, 134.82, 133.81, 132.21, 131.91 (d, *J*_C−F_ = 10.5 Hz), 130.49, 130.44, 129.11, 128.52, 125.86, 125.05 (dd, *J*_C−F_ = 14.2, 3.7 Hz), 120.92, 119.82, 118.50, 111.89 (dd, *J*_C−F_ = 22.1, 3.2 Hz), 105.84 (t, *J*_C−F_ = 25.6 Hz), 53.42, 50.44, 28.45. HRMS (ESI) m/z calcd for C_27_H_23_F_2_N_4_O_4_S [M + H]^+^ 537.1408, found 537.1402.

##### (E)-N-cyclobutyl-3-(6-(5-((2,4-difluorophenyl)sulfonamido)-6-methoxypyridin-3-yl)quinolin-4-yl)acrylamide(8e)

The title compound was prepared from **6e** (53 mg, 0.16 mmol) and **7** (68 mg, 0.16 mmol) according to the general synthetic procedure B, as an off-white solid (25 mg, 0.045 mmol; yield 28%). ^1^H NMR (500 MHz, DMSO-*d*_6_): δ 10.35 (s, 1H, NH), 8.94 (d, *J* = 4.5 Hz, 1H, Ar-H), 8.64 (d, *J* = 7.5 Hz, 1H, NH), 8.54 (d, *J* = 2.0 Hz, 1H, Ar-H), 8.34 (d, *J* = 1.5 Hz, 1H, Ar-H), 8.28 (d, *J* = 15.5 Hz, 1H, alkene hydrogen), 8.16 (d, *J* = 9.0 Hz, 1H, Ar-H), 8.08 (m, 2H, Ar-H), 7.82 (m, 1H, Ar-H), 7.71 (d, *J* = 4.5 Hz, 1H, Ar-H), 7.60 (m, 1H, Ar-H), 7.25 (m, 1H, Ar-H), 6.85 (d, *J* = 15.5 Hz, 1H, alkene hydrogen), 4.44–4.30 (m, 1H, CH), 3.70 (s, 3H, OCH_3_), 2.26 (m, 2H, CH_2_), 1.98 (m, 2H, CH_2_), 1.71 (m, 2H, CH_2_). ^13^C NMR (100 MHz, DMSO-*d*_6_): δ 165.08 (dd, *J*_C−F_ = 253.9, 11.6 Hz), 163.14, 159.34 (dd, *J*_C−F_ = 257.5, 13.5 Hz), 157.57, 150.51, 147.51, 142.85, 140.73, 134.85, 133.97, 133.10, 131.88 (d, *J*_C−F_ = 10.1 Hz), 130.50, 129.13, 129.11, 128.53, 125.82, 125.09 (dd, *J*_C−F_ = 14.8, 3.3 Hz), 120.94, 119.82, 118.63, 111.86 (dd, *J*_C−F_ = 22.5, 3.7 Hz), 105.81 (t, *J*_C−F_ = 25.8 Hz), 53.42, 44.18, 30.27, 14.80. HRMS (ESI) m/z calcd for C_28_H_25_F_2_N_4_O_4_S [M + H]^+^ 551.1564, found 551.1571.

##### (E)-2,4-difluoro-N-(2-methoxy-5-(4-(3-oxo-3-(pyrrolidin-1-yl)prop-1-en-1-yl)quinolin-6-yl)pyridin-3-yl)benzenesulfonamide(8f)

The title compound was prepared from **6f** (53 mg, 0.16 mmol) and **7** (68 mg, 0.16 mmol) according to the general synthetic procedure B, as an off-white solid (21 mg, 0.038 mmol; yield 24%). ^1^H NMR (500 MHz, DMSO-*d*_6_): δ 10.37 (s, 1H, NH), 8.94 (d, *J* = 4.0 Hz, 1H, Ar-H), 8.52 (s, 1H, Ar-H), 8.34 (d, *J* = 15.5 Hz, 1H, alkene hydrogen), 8.33 (brs, 1H, Ar-H), 8.16 (d, *J* = 8.5 Hz, 1H, Ar-H), 8.08 (m, 2H, Ar-H), 7.91 (d, *J* = 4.0 Hz, 1H, Ar-H), 7.83 (m, 1H, Ar-H), 7.60 (m, 1H, Ar-H), 7.28 (d, *J* = 15.5 Hz, 1H, alkene hydrogen), 7.27 (m, 1H, Ar-H), 3.79–3.63 (m, 5H, OCH_3_ + CH_2_), 3.48 (t, *J* = 6.5 Hz, 2H, CH_2_), 1.94 (m, 2H, CH_2_), 1.89–1.81 (m, 2H, CH_2_). ^13^C NMR (100 MHz, DMSO-*d*_6_): δ 165.09 (dd, *J*_C−F_ = 254.5, 10.5 Hz), 162.87, 159.34 (dd, *J*_C−F_ = 257.7, 13.2 Hz), 157.52, 150.39, 147.50, 142.76, 140.65, 134.81, 134.15, 133.78, 131.90 (d, *J*_C−F_ = 11.0 Hz), 130.49, 129.07, 128.39, 127.15, 125.86, 125.07 (dd, *J*_C−F_ = 14.5, 2.4 Hz), 120.80, 119.84, 119.03, 111.88 (dd, *J*_C−F_ = 22.5, 2.6 Hz), 105.80 (t, *J*_C−F_ = 26.6 Hz), 53.40, 46.18, 45.77, 25.60, 23.84. HRMS (ESI) m/z calcd for C_28_H_25_F_2_N_4_O_4_S [M + H]^+^ 551.1564, found 551.1568.

##### (E)-2,4-difluoro-N-(2-methoxy-5-(4-(3-oxo-3-(piperidin-1-yl)prop-1-en-1-yl)quinolin-6-yl)pyridin-3-yl)benzenesulfonamide(8g)

The title compound was prepared from **6g** (55 mg, 0.16 mmol) and **7** (68 mg, 0.16 mmol) according to the general synthetic procedure B, as an off-white solid (12 mg, 0.021 mmol; yield 13%). ^1^H NMR (500 MHz, DMSO-*d*_6_): δ 10.36 (s, 1H, NH), 8.94 (d, *J* = 4.5 Hz, 1H, Ar-H), 8.53 (d, *J* = 2.5 Hz, 1H, Ar-H), 8.35 (s, 1H, Ar-H), 8.33 (d, *J* = 15.5 Hz, 1H, alkene hydrogen), 8.15 (d, *J* = 8.5 Hz, 1H, Ar-H), 8.11–8.05 (m, 2H, Ar-H), 7.96 (d, *J* = 4.5 Hz, 1H, Ar-H), 7.82 (m, 1H, Ar-H), 7.63–7.58 (m, 1H, Ar-H), 7.55 (d, *J* = 15.5 Hz, 1H, alkene hydrogen), 7.26 (m, 1H, Ar-H), 3.69 (s, 5H, OCH_3_ + CH_2_), 3.63–3.56 (m, 2H, CH_2_), 1.64 (m, 2H, CH_2_), 1.55 (m, 4H, CH_2_ × 2). ^13^C NMR (100 MHz, DMSO-*d*_6_): δ 165.09 (dd, *J*_C−F_ = 252.6, 12.1 Hz), 163.75, 159.35 (dd, *J*_C−F_ = 257.7, 13.0 Hz), 157.48, 150.33, 147.53, 142.70, 140.62, 134.78, 134.58, 133.63, 131.89 (d, *J*_C−F_ = 10.8 Hz), 130.48, 129.09, 128.32, 125.96, 125.85, 125.11 (dd, *J*_C−F_ = 14.5, 4.1 Hz), 120.74, 119.89, 118.87, 111.85 (dd, *J*_C−F_ = 22.2, 3.0 Hz), 105.77 (t, *J*_C−F_ = 26.0 Hz), 53.38, 46.34, 42.63, 26.44, 25.35, 24.06. HRMS (ESI) m/z calcd for C_29_H_27_F_2_N_4_O_4_S [M + H]^+^ 565.1721, found 565.1725.

##### (E)-2,4-difluoro-N-(2-methoxy-5-(4-(3-morpholino-3-oxoprop-1-en-1-yl)quinolin-6-yl)pyridin-3-yl)benzenesulfonamide(8h)

The title compound was prepared from **6h** (55 mg, 0.16 mmol) and **7** (68 mg, 0.16 mmol) according to the general synthetic procedure B, as an off-white solid (19 mg, 0.034 mmol; yield 21%). ^1^H NMR (500 MHz, DMSO-*d*_6_): δ 10.36 (s, 1H, NH), 8.95 (d, *J* = 4.5 Hz, 1H, Ar-H), 8.53 (d, *J* = 2.0 Hz, 1H, Ar-H), 8.39 (d, *J* = 15.5 Hz, 1H, alkene hydrogen), 8.35 (d, *J* = 1.5 Hz, 1H, Ar-H), 8.15 (d, *J* = 8.5 Hz, 1H, Ar-H), 8.10–8.05 (m, 2H, Ar-H), 7.95 (d, *J* = 4.5 Hz, 1H, Ar-H), 7.82 (m, 1H, Ar-H), 7.62–7.57 (m, 1H, Ar-H), 7.54 (d, *J* = 15.5 Hz, 1H, alkene hydrogen), 7.25 (td, *J* = 8.5, 2.5 Hz, 1H, Ar-H), 3.77 (m, 2H, CH_2_), 3.69 (s, 3H, OCH_3_), 3.65 (s, 6H, CH_2_ × 3). ^13^C NMR (100 MHz, DMSO-*d*_6_): δ 165.07 (dd, *J*_C−F_ = 253.9, 12.1 Hz), 164.03, 159.34 (dd, *J*_C−F_ = 256.5, 14.3 Hz), 157.57, 150.36, 147.53, 142.84, 140.49, 135.29, 134.81, 133.98, 131.88 (d, *J*_C−F_ = 10.7 Hz), 130.49, 129.08, 128.41, 125.83, 125.24, 125.10 (dd, *J*_C−F_ = 14.6, 3.7 Hz), 120.80, 119.83, 118.97, 111.86 (dd, *J*_C−F_ = 22.1, 3.4 Hz), 105.79 (t, *J*_C−F_ = 26.2 Hz), 66.36, 66.11, 53.40, 45.82, 42.15. HRMS (ESI) m/z calcd for C_28_H_25_F_2_N_4_O_5_S [M + H]^+^ 567.1513, found 567.1514.

##### (E)-2,4-difluoro-N-(2-methoxy-5-(4-(3-(4-methylpiperazin-1-yl)-3-oxoprop-1-en-1-yl)quinolin-6-yl)pyridin-3-yl)benzenesulfonamide(8i)

The title compound was prepared from **6i** (57 mg, 0.16 mmol) and **7** (68 mg, 0.16 mmol) according to the general synthetic procedure B, as an off-white solid (22 mg, 0.038 mmol; yield 24%). ^1^H NMR (500 MHz, DMSO-*d*_6_): δ 10.36 (s, 1H, NH), 8.94 (d, *J* = 4.5 Hz, 1H, Ar-H), 8.49 (d, *J* = 2.5 Hz, 1H, Ar-H), 8.36 (d, *J* = 15.5 Hz, 1H, alkene hydrogen), 8.34 (d, *J* = 2.0 Hz, 1H, Ar-H), 8.15 (d, *J* = 8.5 Hz, 1H, Ar-H), 8.08 (dd, *J* = 8.5, 2.0 Hz, 1H, Ar-H), 8.05 (d, *J* = 2.5 Hz, 1H), 7.96 (d, *J* = 4.5 Hz, 1H, Ar-H), 7.83 (m, 1H, Ar-H), 7.58 (m, 1H, Ar-H), 7.58 (d, *J* = 15.5 Hz, 1H, alkene hydrogen), 7.25 (td, *J* = 8.5, 2.5 Hz, 1H, Ar-H), 3.76 (m, 2H, CH_2_), 3.70 (s, 3H, OCH_3_), 3.65 (m, 2H, CH_2_), 2.45–2.35 (m, 4H, CH_2_ × 2), 2.25 (s, 3H, CH_3_). HRMS (ESI) m/z calcd for C_29_H_28_F_2_N_5_O_4_S [M + H]^+^ 580.1830, found 580.1819.

##### (E)-2,4-difluoro-N-(5-(4-(3-(4-isopropylpiperazin-1-yl)-3-oxoprop-1-en-1-yl)quinolin-6-yl)-2-methoxypyridin-3-yl)benzenesulfonamide(8j)

The title compound was prepared from **6j** (62 mg, 0.16 mmol) and **7** (68 mg, 0.16 mmol) according to the general synthetic procedure B, as an off-white solid (25 mg, 0.041 mmol; yield 26%). ^1^H NMR (500 MHz, DMSO-*d*_6_): δ 10.34 (s, 1H, NH), 8.94 (d, *J* = 4.5 Hz, 1H, Ar-H), 8.48 (s, 1H), 8.35 (d, *J* = 15.5 Hz, 2H, Ar-H + alkene hydrogen), 8.15 (d, *J* = 9.0 Hz, 1H, Ar-H), 8.11–8.03 (m, 2H, Ar-H), 7.95 (d, *J* = 4.5 Hz, 1H, Ar-H), 7.82 (m, 1H, Ar-H), 7.56 (m, 2H, Ar-H + alkene hydrogen), 7.24 (m, 1H, Ar-H), 3.74 (m, 2H, CH_2_), 3.69 (s, 3H, OCH_3_), 3.64 (m, 2H, CH_2_), 2.74 (m, 1H, CH), 2.52 (m, 4H, CH_2_ × 2), 1.01 (s, 3H, CH_3_), 0.99 (s, 3H, CH_3_). ^13^C NMR (100 MHz, DMSO-*d*_6_): δ 167.39 (dd, *J*_C−F_ = 242.7, 13.7 Hz), 163.75, 159.31 (dd, *J*_C−F_ = 255.8, 14.5 Hz), 157.52, 150.33, 147.50, 142.10, 140.52, 135.09, 134.94, 133.20, 131.86 (d, *J*_C−F_ = 11.0 Hz), 130.47, 129.01, 128.40, 125.82, 125.52, 120.70, 118.96, 111.98–111.58 (dd, *J*_C−F_ = 22.2, 4.4 Hz), 105.72 (t, *J*_C−F_ = 25.3 Hz), 53.89, 53.32, 48.66, 47.76, 45.47, 41.90, 17.90. HRMS (ESI) m/z calcd for C_31_H_32_F_2_N_5_O_4_S [M + H]^+^ 608.2143, found 608.2135.

##### (E)-2,4-difluoro-N-(5-(4-(3-(4-hydroxypiperidin-1-yl)-3-oxoprop-1-en-1-yl)quinolin-6-yl)-2-methoxypyridin-3-yl)benzenesulfonamide(8k)

The title compound was prepared from **6k** (58 mg, 0.16 mmol) and **7** (68 mg, 0.16 mmol) according to the general synthetic procedure B, as an off-white solid (25 mg, 0.043 mmol; yield 27%). ^1^H NMR (500 MHz, DMSO-*d*_6_): δ 10.36 (s, 1H, NH), 8.94 (d, *J* = 4.5 Hz, 1H, Ar-H), 8.54 (d, *J* = 2.0 Hz, 1H, Ar-H), 8.34 (d, *J* = 15.0 Hz, 1H, alkene hydrogen), 8.36 (d, *J* = 1.5 Hz, 1H, Ar-H), 8.15 (d, *J* = 8.5 Hz, 1H, Ar-H), 8.10–8.06 (m, 2H, Ar-H), 7.97 (d, *J* = 4.5 Hz, 1H, Ar-H), 7.85–7.79 (m, 1H, Ar-H), 7.64–7.59 (m, 1H, Ar-H), 7.57 (d, *J* = 15.0 Hz, 1H, alkene hydrogen), 7.25 (td, *J* = 8.5, 2.0 Hz, 1H, Ar-H), 4.80 (brs, 1H, OH), 4.04 (m, 2H, CH_2_), 3.77 (m, 1H, CH), 3.69 (s, 3H, OCH_3_), 3.45–3.39 (m, 1H, CH_2_), 3.24 (m, 1H, CH_2_), 1.79 (m, 2H, CH_2_), 1.44–1.35 (m, 2H, CH_2_). ^13^C NMR (100 MHz, DMSO-*d*_6_): δ 165.14 (dd, *J*_C−F_ = 252.7, 11.8 Hz), 163.78, 159.35 (dd, *J*_C−F_ = 257.5, 13.5 Hz), 157.56, 150.37, 147.53, 142.88, 140.59, 134.77, 133.98, 131.89 (d, *J*_C−F_ = 10.8 Hz), 130.48, 129.11, 128.39, 125.87, 125.85, 125.06 (dd, *J*_C−F_ = 14.5, 3.8 Hz), 120.80, 119.76, 118.91, 111.87 (dd, *J*_C−F_ = 22.1, 3.4 Hz), 105.80 (t, *J*_C−F_ = 26.1 Hz), 65.45, 53.40, 42.83, 41.28, 34.94, 33.90. HRMS (ESI) m/z calcd for C_29_H_27_F_2_N_4_O_5_S [M + H]^+^ 581.1670, found 581.1666.

##### (E)-2,4-difluoro-N-(5-(4-(3-(3-hydroxypiperidin-1-yl)-3-oxoprop-1-en-1-yl)quinolin-6-yl)-2-methoxypyridin-3-yl)benzenesulfonamide(8l)

The title compound was prepared from **6l** (58 mg, 0.16 mmol) and **7** (68 mg, 0.16 mmol) according to the general synthetic procedure B, as an off-white solid (21 mg, 0.036 mmol; yield 23%). ^1^H NMR (500 MHz, DMSO-*d*_6_): δ 10.35 (s, 1H, NH), 8.95 (d, *J* = 4.5 Hz, 1H, Ar-H), 8.53 (brs, 1H, Ar-H), 8.33 (d, *J* = 14.0 Hz, 2H, Ar-H + alkene hydrogen), 8.15 (d, *J* = 8.5 Hz, 1H, Ar-H), 8.08 (brd, *J* = 8.0 Hz, 2H, Ar-H), 8.03–7.92 (m, 1H, Ar-H), 7.83 (m, 1H, Ar-H), 7.63–7.49 (m, 2H, Ar-H + alkene hydrogen), 7.25 (m, 1H, Ar-H), 4.99 (d, *J* = 4.5 Hz, 0.52H, OH), 4.88 (d, *J* = 4.0 Hz, 0.51H, OH), 4.30 (m, 0.53H, CH), 4.02 (m, 0.53H, CH), 3.83 (m, 0.57H, CH_2_), 3.69 (s, 2H, OCH_3_), 3.62 (m, 1H, CH_2_), 3.57 (s, 1H, OCH_3_), 3.45 (m, 1.56H, CH_2_), 3.22 (m, 0.63H, CH_2_), 2.84–2.72 (m, 0.52H, CH_2_), 1.84 (m, 2H, CH_2_), 1.41 (m, 2H, CH_2_). ^13^C NMR (100 MHz, DMSO-*d*_6_): δ 165.55 (dd, *J*_C−F_ = 254.3, 11.7 Hz), 164.80, 164.46, 159.84 (dd, *J*_C−F_ = 257.6, 13.5 Hz), 158.04, 150.87, 148.02, 143.21, 141.19, 141.07, 135.30, 134.89, 134.31, 132.40 (d, *J*_C−F_ = 10.5 Hz), 130.98, 130.12, 130.07, 128.90, 126.65, 126.46, 126.34, 125.63 (dd, *J*_C−F_ = 14.3, 2.7 Hz), 121.27, 120.46, 119.43, 119.28, 112.35 (dd, *J*_C−F_ = 22.0, 3.2 Hz), 106.29 (t, *J*_C−F_ = 26.1 Hz), 66.82, 65.65, 53.89, 52.90, 49.53, 45.98, 42.65, 33.54, 32.74, 25.40, 25.07, 24.36, 22.27. HRMS (ESI) m/z calcd for C_29_H_27_F_2_N_4_O_5_S [M + H]^+^ 581.1670, found 581.1668.

##### (E)-2,4-difluoro-N-(5-(4-(3-(4-(hydroxymethyl)piperidin-1-yl)-3-oxoprop-1-en-1-yl)quinolin-6-yl)-2-methoxypyridin-3-yl)benzenesulfonamide(8m)

The title compound was prepared from **6m** (60 mg, 0.16 mmol) and **7** (68 mg, 0.16 mmol) according to the general synthetic procedure B, as an off-white solid (28 mg, 0.047 mmol; yield 29%). ^1^H NMR (500 MHz, DMSO-*d*_6_): δ 10.35 (s, 1H, NH), 8.93 (d, *J* = 4.5 Hz, 1H, Ar-H), 8.54 (d, *J* = 2.0 Hz, 1H, Ar-H), 8.39–8.31 (m, 2H, Ar-H + alkene hydrogen), 8.15 (d, *J* = 9.0 Hz, 1H, Ar-H), 8.11–8.05 (m, 2H, Ar-H), 7.96 (d, *J* = 4.5 Hz, 1H, Ar-H), 7.81 (m, 1H, Ar-H), 7.57 (m, 2H, Ar-H + alkene hydrogen), 7.25 (m, 1H, Ar-H), 4.53 (m, 2H, CH_2_), 4.31 (d, *J* = 12.5 Hz, 1H, OH), 3.69 (s, 3H, OCH_3_), 3.28 (m, 2H, CH_2_), 3.13 (m, 1H, CH_2_), 2.71 (m, 1H, CH_2_), 1.76 (m, 3H, CH + CH_2_), 1.13 (m, 2H, CH_2_). ^13^C NMR (100 MHz, DMSO-*d*_6_): δ 165.07 (dd, *J*_C−F_ = 254.4, 12.2 Hz), 163.74, 159.34 (dd, *J*_C−F_ = 257.6, 13.1 Hz), 157.55, 150.38, 147.52, 142.82, 140.61, 134.77, 134.72, 133.93, 131.89 (d, *J*_C−F_ = 11.9 Hz), 130.48, 129.09, 128.39, 125.96, 125.84, 125.07 (dd, *J*_C−F_ = 14.3, 4.5 Hz), 120.80, 119.82, 118.92, 111.86 (dd, *J*_C−F_ = 22.0, 3.4 Hz), 105.80 (t, *J*_C−F_ = 25.5 Hz), 65.46, 53.40, 45.35, 41.77, 38.41, 29.55, 28.42. HRMS (ESI) m/z calcd for C_30_H_29_F_2_N_4_O_5_S [M + H]^+^ 595.1827, found 595.1820.

##### (E)-3-(6-(5-((2,4-difluorophenyl)sulfonamido)-6-methoxypyridin-3-yl)quinolin-4-yl)-N-(2-morpholinoethyl)acrylamide(8n)

The title compound was prepared from **6n** (62 mg, 0.16 mmol) and **7** (68 mg, 0.16 mmol) according to the general synthetic procedure B, as an off-white solid (15 mg, 0.025 mmol; yield 16%). ^1^H NMR (500 MHz, DMSO-*d*_6_): δ 10.32 (s, 1H, NH), 8.94 (d, *J* = 4.5 Hz, 1H, Ar-H), 8.51 (brs, 1H, NH), 8.35 (m, 2H, Ar-H), 8.28 (d, *J* = 15.5 Hz, 1H, alkene hydrogen), 8.15 (d, *J* = 9.0 Hz, 1H, Ar-H), 8.07 (m, 2H, Ar-H), 7.82 (m, 1H, Ar-H), 7.73 (d, *J* = 4.5 Hz, 1H, Ar-H), 7.59 (m, 1H, Ar-H), 7.25 (m, 1H, Ar-H), 6.95 (d, *J* = 15.5 Hz, 1H, alkene hydrogen), 3.70 (s, 3H, OCH_3_), 3.59 (m, 4H, CH_2_ × 2), 3.39 (m, 2H, CH_2_), 2.44 (m, 6H, CH_2_ × 3). HRMS (ESI) m/z calcd for C_30_H_30_F_2_N_5_O_5_S [M + H]^+^ 610.1936, found 610.1913.

##### (E)-3-(6-(5-((2,4-difluorophenyl)sulfonamido)-6-methoxypyridin-3-yl)quinolin-4-yl)-N-phenylacrylamide(8o)

The title compound was prepared from **6o** (56 mg, 0.16 mmol) and **7** (68 mg, 0.16 mmol) according to the general synthetic procedure B, as an off-white solid (15 mg, 0.026 mmol; yield 16%). ^1^H NMR (500 MHz, CDCl_3_): δ 10.44 (s, 1H, NH), 10.35 (s, 1H, NH), 8.97 (d, *J* = 4.5 Hz, 1H, Ar-H), 8.51 (brs, 1H, Ar-H), 8.46 (d, *J* = 15.5 Hz, 1H, alkene hydrogen), 8.40 (brs, 1H, Ar-H), 8.17 (d, *J* = 9.0 Hz, 1H, Ar-H), 8.09 (d, *J* = 9.0 Hz, 2H, Ar-H), 7.78 (m, 4H, Ar-H), 7.57 (m, 1H, Ar-H), 7.37 (t, *J* = 8.0 Hz, 2H, Ar-H), 7.24 (m, 1H, Ar-H), 7.15–7.07 (m, 2H, Ar-H + alkene hydrogen), 3.68 (s, 3H, OCH_3_). ^13^C NMR (100 MHz, DMSO-*d*_6_): δ 165.03 (dd, *J*_C−F_ = 254.0, 11.8 Hz), 162.82, 159.35 (dd, *J*_C−F_ = 257.3, 13.7 Hz), 157.60, 150.50, 147.56, 142.72, 140.47, 138.98, 135.00, 134.50, 133.87, 131.86 (d, *J*_C−F_ = 10.6 Hz), 130.51, 129.23, 129.08, 128.83, 128.58, 125.82, 125.23 (dd, *J*_C−F_ = 13.5, 4.9 Hz), 123.69, 120.97, 120.09, 119.42, 118.75, 111.81 (dd, *J*_C−F_ = 22.1, 3.1 Hz), 105.76 (t, *J*_C−F_ = 26.0 Hz), 53.39. HRMS (ESI) m/z calcd for C_30_H_23_F_2_N_4_O_4_S [M + H]^+^ 573.1408, found 573.1402.

##### 2,4-Difluoro-N-(2-methoxy-5-(quinolin-6-yl)pyridin-3-yl)benzenesulfonamide(10)

The title compound was prepared from 6-bromoquinoline (25 mg, 0.12 mmol) and **7** (50 mg, 0.12 mmol) according to the general synthetic procedure B as an off-white solid (23 mg, 0.054 mmol; yield 45%). ^1^H NMR (500 MHz, DMSO-*d*_6_): δ 10.36 (s, 1H, NH), 8.93 (dd, *J* = 4.0, 1.5 Hz, 1H, Ar-H), 8.51 (d, *J* = 2.0 Hz, 1H, Ar-H), 8.45 (dd, *J* = 8.5, 1.0 Hz, 1H, Ar-H), 8.27 (d, *J* = 2.0 Hz, 1H, Ar-H), 8.12 (d, *J* = 9.0 Hz, 1H, Ar-H), 8.06 (m, 2H, Ar-H), 7.79 (m, 1H, Ar-H), 7.62–7.56 (m, 2H, Ar-H), 7.23 (m, 1H, Ar-H), 3.68 (s, 3H, OCH3). ^13^C NMR (125 MHz, DMSO-*d*_6_): δ 165.56 (dd, *J*_C−F_ = 252.5, 11.8 Hz), 159.88 (dd, *J*_C−F_ = 256.1, 13.4 Hz), 158.13, 151.23, 147.58, 143.16, 136.73, 134.78, 134.57, 132.36 (d, *J*_C−F_ = 16.0 Hz), 130.24, 129.37, 128.65, 128.56, 126.65, 125.56 (dd, *J*_C−F_ = 13.8, 3.6 Hz), 122.46, 120.22, 112.31 (dd, *J*_C−F_ = 22.1, 3.1 Hz), 106.27 (t, *J*_C−F_ = 25.8 Hz), 53.90. ESI-MS: m/z = 428 [M+H] ^+^.

### Biology

#### In vitro Enzymatic and Anti-proliferative Assays

Kinase-Glo Luminescent Kinase assay (Promega) was performed to determine the PI3Kα inhibitory activity, while ADP-Glo Luminescent assay (Promega) was performed to identify the inhibitory activity against PI3Kβ, γ, or δ. The kinase buffer was composed of HEPES (50 mM, pH 7.5), MgCl_2_ (3 mM), EGTA (1 mM), NaCl (100 mM), CHAPS (0.03%) and DTT (2 mM). The serially diluted compound solutions (2.5 mL) were successively added to a 384-well plate. Afterwards, the solution of kinase (2.5 mL) in the kinase buffer and the solution of substrate (5 mL) in the kinase buffer containing PIP_2_ and ATP were added to each well of the assay plate for starting the reaction. Kinase buffer was added instead to the control well without enzyme. The final concentrations of PI3Kα, PI3Kβ, PI3Kγ, PI3Kδ, PIP_2_, and ATP in corresponding kinase reaction mixture were 1.65 nM, 4.8 nM, 7.6 nM, 5.7 nM, 50 mM and 25 mM, respectively. After being incubated at room temperature for 1 h, Kinase-Glo reagent (10 mL) was added to each well of the PI3Kα assay plate to quench the reaction. Subsequently, the mixture needs to be mixed briefly with centrifuge and shaken slowly for 15 min before being read on a plate reader for luminescence. As for PI3Kβ, γ, or δ, reaction mixture (5 mL) was transferred to a new 384-plate and ADP-Glo reagent (5 mL) was added for stopping the reaction. The mixture was treated briefly with centrifuge, shaken slowly, and equilibrated for 40 min. Kinase Detection reagent (10 mL) was added to each well. Before reading on a plate reader for luminescence, the resultant mixture was incubated at room temperature for 1 h. After calculating the percent inhibition from the RLU values, the curves were fitted in Graphpad Prism 5 to give IC_50_ values.

Lance Ultra assay was performed to determine the mTOR (1362-end, Millipore) inhibitory activity. The kinase buffer was composed of HEPES (50 mM, pH 7.5), MgCl_2_ (10 mM), EGTA (1 mM), MnCl_2_ (3 mM), Tween-20 (0.01%) and DTT (2 mM). The solution of substrate in the kinase buffer contained ULight-4E-BP1 peptide (Thr37/46, PE) and ATP. The kinase reaction mixture was prepared in a similar way to that for class I PI3K inhibition assays. The final concentrations of mTOR, ULight-4E-BP1 peptide and ATP in the kinase reaction mixture were 2.5 nM, 50 mM and 10.8 mM, respectively. After being incubated at room temperature for 1 h, kinase detection buffer (10 mL) containing EDTA and Eu-anti-phospho-4E-BP1 antibody (Thr37/46, PE) was added to each well of the assay plate. Before reading on a plate reader, the mixture needs to be mixed briefly with centrifuge and then allowed to equilibrate for 1 h. After calculating the percent inhibition from Lance signal, the curves were fitted in Graphpad Prism 5 to give IC_50_ values.

The anti-proliferative activity of compounds was evaluated by sulforhodamin B (SRB) assay. After being cultured for 10 h, cells in 96-well plates were subjected to 72 h-exposure to serially diluted compound solutions. Cells were washed with PBS and fixed with trichloroacetic acid (10%, W/V) at 4°C for 1 h. Then, cells were successively washed with PBS, stained for 0.5 h with SRB (0.4%, dissolved in 1% acetic acid), and washed by 1% acetic acid for 5 times. Protein-bound dye was extracted with unbuffered Tris base (10 mmol). After calculating the percent inhibition from absorbance, measured at 515 nm with a multiscan spectrum (Thermo Electron Co., Vantaa, Finland), the curves were fitted in Graphpad Prism 5 to give GI_50_ values.

#### Western Blot Assay

The Western blot analysis for evaluating the capability to down-regulate phos-Akt (Ser473), phos-Akt (Thr308), phos-S6 ribosomal protein (Ser235/236), and phos-4E-BP1 (Thr37/46) in PC3 cells was performed according to the protocol disclosed in our previous study (Zhang et al., 2017a) with a minor modification. The cells were seeded into six-well plate at 1 × 10^6^ cells per well, and then incubated at 37°C (5% CO_2_) overnight prior to drug treatment. Cells were treated with **8i** and GSK2126458 at various concentrations and incubated at 37°C for 3 h.

#### Liver Microsomal Stability Assay

The HLM and SD rat RLM utilized for this experiment were purchased from Corning and Xenotech, respectively. An appropriate amount of NADPH powder was weighed and dissolved in MgCl_2_ solution (10 mM). The microsome working solutions were prepared with potassium phosphate buffer (100 mM). Cold acetonitrile containing tolbutamide (100 ng/mL) and labetalol (100 ng/mL) as internal standard was used as the stop solution. Compound or control working solution (10 μL/well) was added to all plates (T0, T5, T10, T20, T30, T60, NCF60) except matrix blank. After microsome solution was dispensed to every plate (80 μL/well), the mixture was incubated at 37°C for about 10 min. Subsequently, potassium phosphate buffer (100 mM) was added to the plate NCF60 (10 μL/well), and the mixture was incubated at 37°C. As for other plates, the pre-warmed NADPH solution (10 μL/well) was added to start the reaction. At each time point, the stop solution (precooled in 4°C) was added to terminate the reaction (300 μL/well). After shaking and centrifuging, 100 μL supernatant was mixed with an appropriate amount of water for LC/MS/MS analysis.

#### Pharmacokinetic (PK) Study

SD rats were utilized for the PK study of **8i** following oral gavage at the dosage of 5 mg/kg, and the oral dose was formulated in a homogenous opaque suspension of 0.5% methylcellulose. This experiment was performed with the protocol disclosed in our previous work (Zhang et al., 2017b). The study was carried out in accordance with institutional guidelines of the Animal Research Committee at Jiaxing University (log number JXU2015120812). The protocol was approved by the institution.

#### Molecular Docking Study

The co-crystal structure of PI3Kα in complex with BYL-719 (PDB code 4JPS) was selected as the template for the molecular docking analysis, which was conducted using Discovery Studio (Version 2.1). For preparing the ligands, 3D structures were generated, and their energy minimization performed. For preparing the receptor, the hydrogen atoms were added, and the CHARMm-force field was employed. The site sphere was chosen based on the binding location of BYL-719 in PI3Kα. BYL-719 was removed and **8i**, as the ligand, was docked into the defined site. After analyzing the types of interactions between **8i** and PI3Kα, the final binding conformation was determined according to the calculated energy.

## Results and Discussion

### Chemistry

**Scheme 1:**
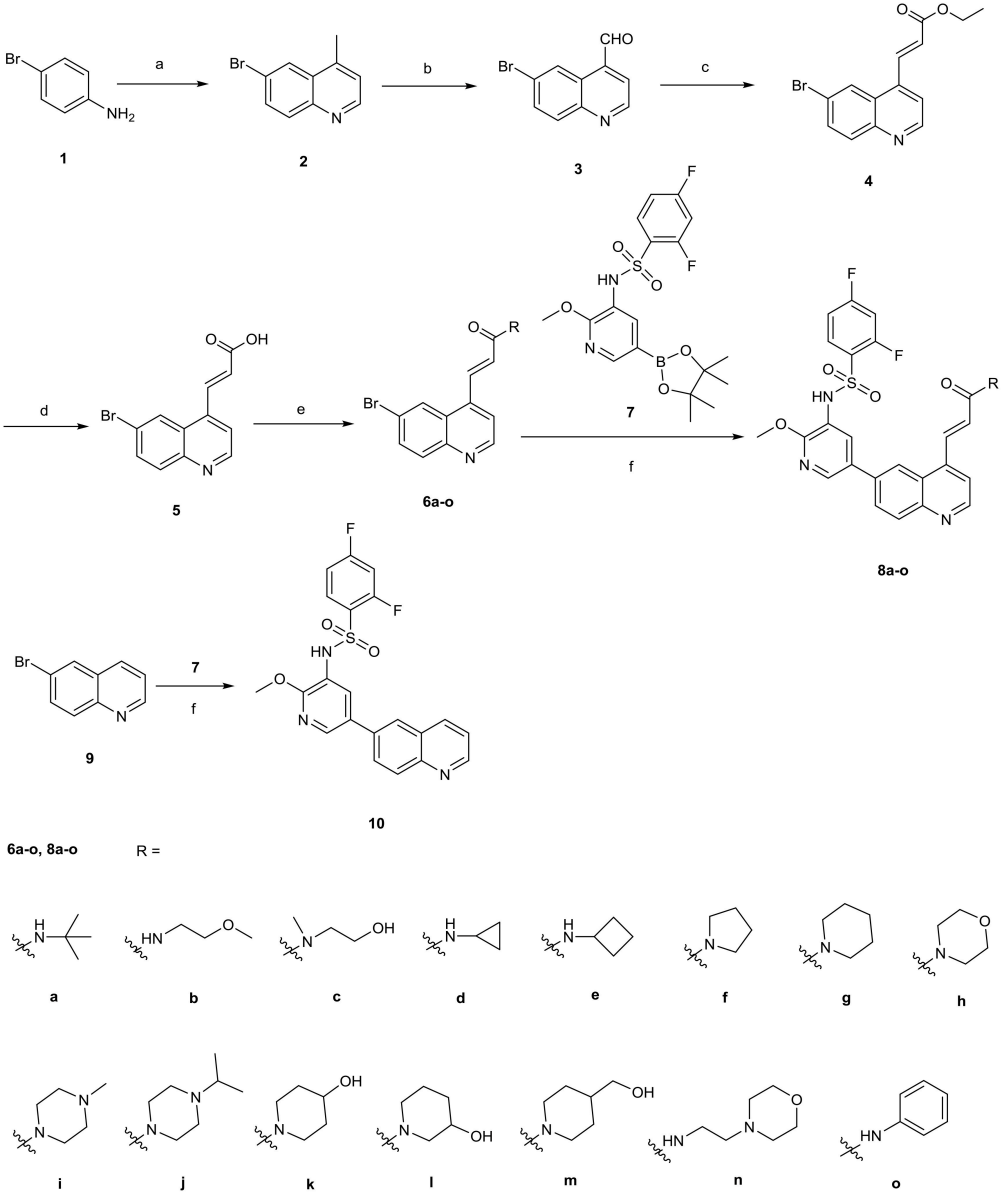
The synthetic route for target compounds **8a-o** and **10**. Reagents and conditions: **(a)** methyl vinyl ketone, FeCl_3_, CH_3_COOH, 70°C, 3 h; ZnCl_2_, reflux, 2 h; **(b)** SeO_2_, dioxane/H_2_O, 100°C, 2 h; **(c)** NaH, triethyl phosphonoacetate, THF, 0°C to rt, 1.5 h; **(d)** 2.5 N NaOH, rt, 2 h; 2 N HCl; **(e)** EDCI, HOBt, DCM, rt, 2 h; corresponding amine, TEA, 1 h, rt; **(f)** Pd(dppf)_2_Cl_2_, K_2_CO_3_, dioxane/H_2_O, 100°C, 10 h.

The synthetic route for 4-acrylamido-quinolines **8a**–**o** and C-4 unsubstituted quinoline derivative **10** is outlined in Scheme 1. The reaction of 4-bromoaniline **1** with methyl vinyl ketone in the presence of FeCl_3_ and ZnCl_2_ provided 4-methyl-6-bromoquinoline **2** (Phanumartwiwath et al., 2013). Following oxidation of **2** with SeO_2_ (Perez-Melero et al., 2004), the newly formed 6-bromoquinoline-4-carbaldehyde **3** was treated with triethyl phosphonoacetate, thereby leading to the generation of ethyl (*E*)-3-(6-bromoquinolin-4-yl)acrylate **4** (Lalonde et al., 2013). Afterwards, hydrolysis of **4** afforded (*E*)-3-(6-bromoquinolin-4-yl)acrylic acid **5**, which was treated with corresponding amine in the presence of EDCI and HOBt to give the quinoline derivatives **6a-o**. Subsequent Suzuki coupling of **6a**–**o** with the borate **7**, which was prepared with a procedure disclosed in our previous work (Lv et al., 2015), yielded corresponding target compounds **8a**–**o**. Similarly, compound **10** was prepared by Suzuki coupling of **9** with **7**.

### Biology

#### *In vitro* PI3Kα Enzymatic and Anti-Proliferative Assays

**Figure 2:**
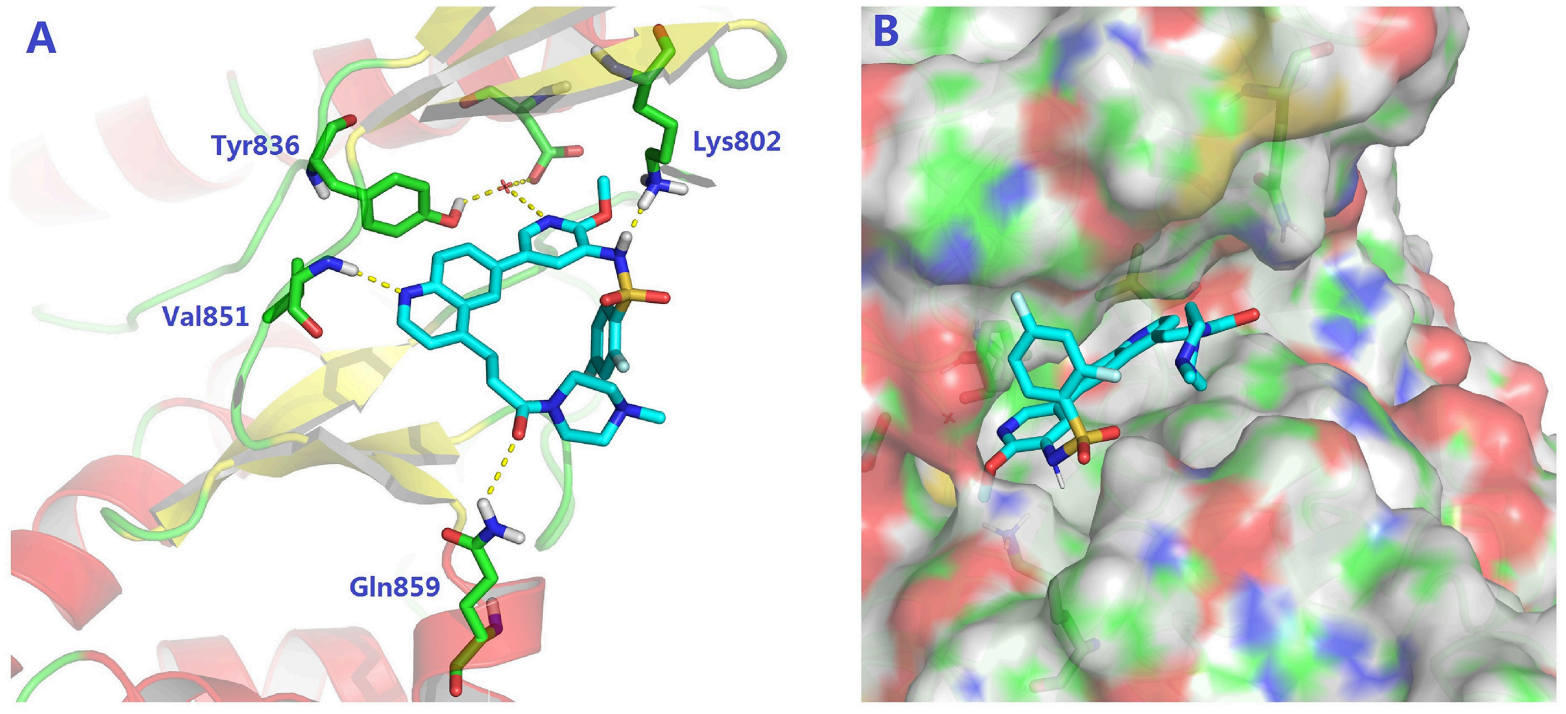
The molecular docking of compound **8i** into PI3Kα, indicating the interaction of C-4 acrylamide substructure with Gln859. **(A)** the binding mode of **8i** with the catalytic site; **(B)** the binding mode with surface area highlighted.

To validate the design rationale for the target compounds, they were evaluated for the PI3Kα inhibitory activity, as well as anti-proliferative efficacy against prostate cancer PC3 and colorectal cancer HCT116 cell lines (Table 1). According to the experimental results of the PI3Kα enzymatic assay, all the quinolines bearing acrylamides as the C-4 replacements exhibited potent PI3Kα inhibitory activity with IC_50_ values below 3 nM. Among them, compound **8i** exerted the most potent PI3Kα inhibitory activity (IC_50_ = 0.5 nM), which was comparable to that of GSK2126458, the clinically investigated PI3K/mTOR dual inhibitor. Importantly, compounds **8a-o** with various C-4 acrylamide substructures displayed over 7-fold improvement in the enzymatic activity compared to the C-4 unsubstituted counterpart **10**, suggesting that the acrylamide replacements played an important role in boosting PI3Kα inhibitory activity. The structure-activity relationship was consistent with our docking analysis, which demonstrated that acrylamide substructure introduced at the C-4 position of the quinoline template had the potential to probe Gln859 *via* H-bond interaction, as exemplified by the modeling of **8i**, a representative throughout this series, into the currently available ATP-binding pocket of PI3Kα (PDB code 4JPS) (Figure 2).

**Table 1:** PI3Kα inhibitory activity and anti-proliferative activity of compounds **8a-o** and **10**.

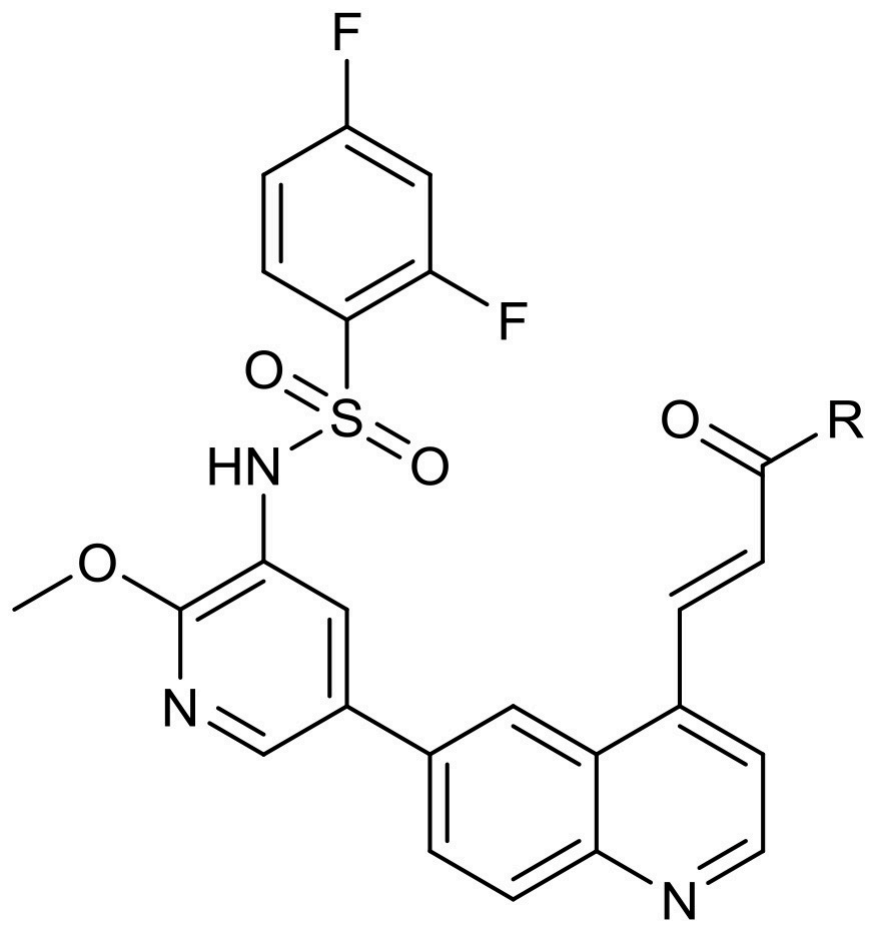				
**Compd**.	**R**	**PI3Kα** **IC**_**50**_ **(nM)**	**GI**_**50**_ **(μM)**	
			**PC3**	**HCT116**
**10**	–	14.28	7.16	3.67
**8a**	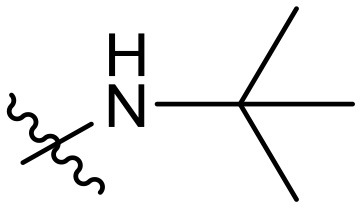	0.63	1.90	0.70
**8b**	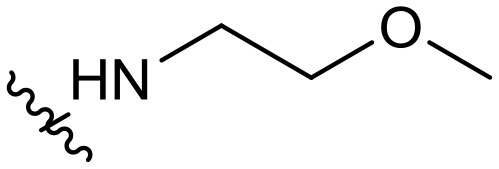	2.03	0.84	1.13
**8c**	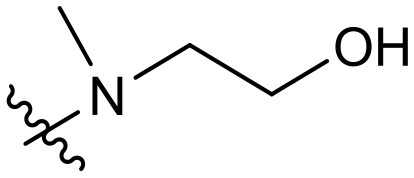	0.67	0.45	0.96
**8d**	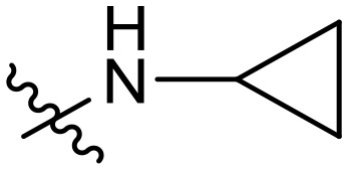	1.03	1.87	1.56
**8e**	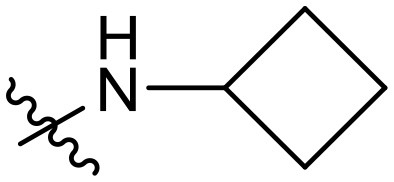	1.43	1.48	1.25
**8f**	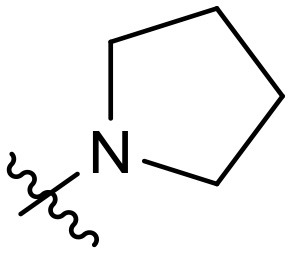	0.62	0.95	1.46
**8g**	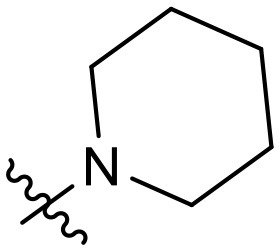	0.68	2.50	1.30
**8h**	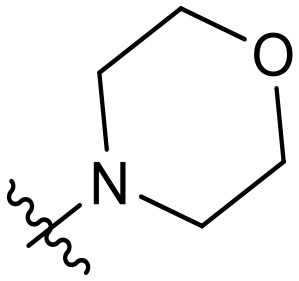	0.53	0.36	0.51
**8i**	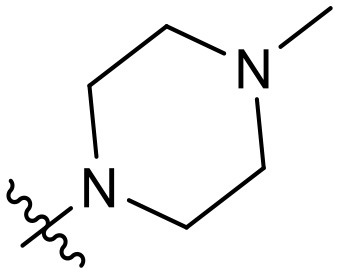	0.50	0.40	0.47
**8j**	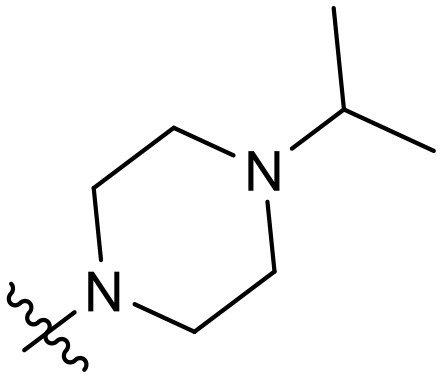	1.90	0.88	0.97
**8k**	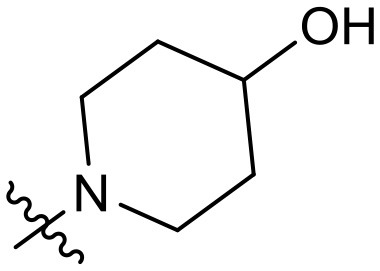	0.79	0.28	0.32
**8l**	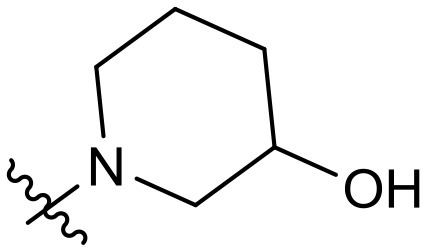	0.75	1.31	1.56
**8m**	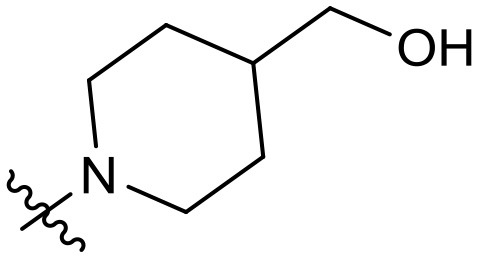	0.68	2.56	1.29
**8n**	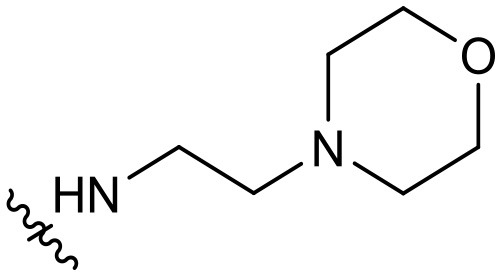	1.10	1.43	1.79
**8o**	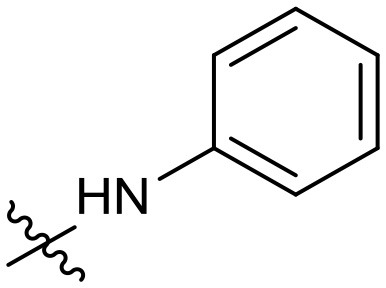	1.80	4.83	1.52
**GSK2126458**	**-**	0.30	0.22	0.18

As illustrated by the results of the anti-proliferative assay (Table 1), all the 4-acrylamido-quinoline derivatives displayed sub-micromolar to low micromolar anti-proliferative activity against both PC3 and HCT116 cell lines. Four compounds in particular (**8c**, **8h**, **8i**, and **8k**) exerted remarkable anti-proliferative activity against PC3 cell line with GI_50_ values below 0.5 μM, which was comparable to that of the positive reference GSK2126458. Besides, they also exhibited sub-micromolar GI_50_ values against HCT116 cell line.

#### Inhibitory Activity Against Other Class I PI3Ks and mTOR

Considering their favorable enzymatic and cellular activities, **8c** and **8i**, as the representatives throughout this series, were then evaluated for the inhibitory activity against other class I PI3Ks and mTOR with GSK2126458 as the positive control. As shown in Table 2, both compounds displayed strong inhibitory activity with IC_50_ values against all the tested enzymes ranging from 0.50 to 1.5 nM. This result suggested that compounds **8c** and **8i** were potent PI3K/mTOR dual inhibitors.

**Table 2:** Enzymatic activity of compounds **8c** and **8i** against PI3Ks and mTOR.

**Compd**.	**IC**_****50****_ **(nM)**				
	**PI3Kα**	**PI3Kβ**	**PI3Kγ**	**PI3Kδ**	**mTOR**
**8c**	0.67	1.2	0.50	1.3	1.2
**8i**	0.50	1.5	0.55	1.0	1.3
**GSK2126458**	0.30	0.19	0.44	0.78	0.34

#### Western Blot Analysis

**Figure 3:**
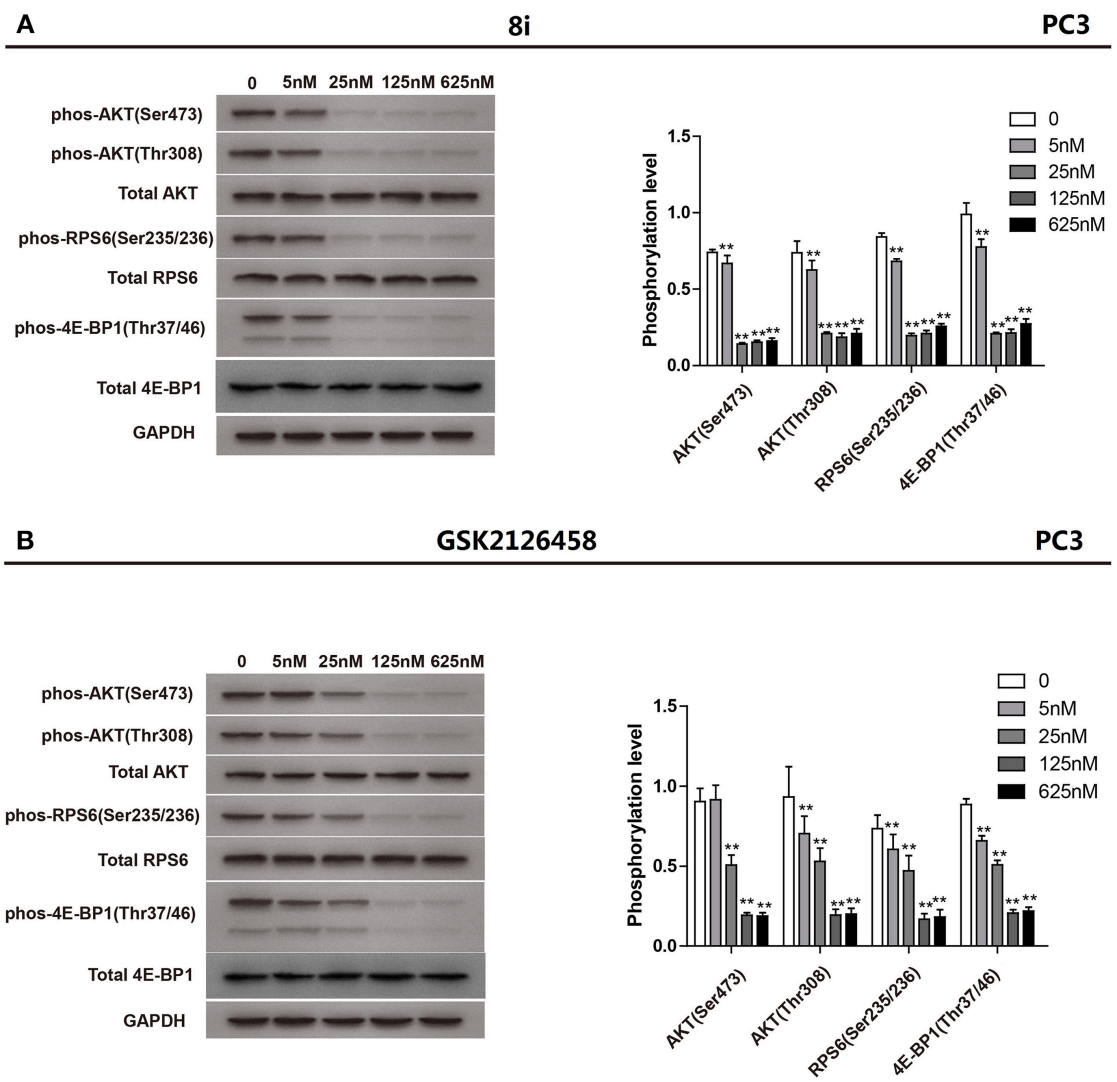
The suppressive effect of **8i (A)** and GSK2126458 **(B)** on the phosphorylation of Akt, S6 Ribosomal protein, and 4E-BP1 in PC3 cells following 3 h-treatment: The levels of phos-Akt (Ser473), phos-Akt (Thr308), phos-S6 ribosomal protein (Ser235/236), and phos-4E-BP1 (Thr37/46) in different groups were determined *via* Western blot assay. The bar chart represented the quantification of the bands in the Western blot with the results shown as mean ± SD (n = 3 biological replicates). **p < 0.01 vs. control (cells incubated without **8i** or GSK2126458).

Subsequently, compound **8i** was investigated for its capability to down-regulate the levels of phos-Akt (Ser473), phos-Akt (Thr308), phos-S6 ribosomal protein (Ser235/236), and phos-4E-BP1 (Thr37/46), four important biomarkers of PAM signaling, in PC3 cells. The suppressive effect of **8i** was evaluated at the concentrations of 5, 25, 125, and 625 nM. As illustrated in Figure 3, at the concentration as low as 5 nM, **8i** remarkably down-regulated all the four monitored biomarkers. In particular, the effect on the level of phos-Akt (Ser473) was stronger than that of GSK2126458. These results further demonstrated **8i** can strongly down-regulate PAM signaling. Moreover, the concurrent down-regulation of the four biomarkers indicated the potential to fulfill synergism, as well as the advantage over mono-inhibition of mTOR in conquering the negative feedback loop.

#### Liver Microsomal Stability Assay

The evaluation of the *in vitro* metabolic stability of **8i** was then performed *via* incubation with human liver microsome (HLM) and rat liver microsome (RLM). Testosterone, diclofenac, and propafenone were utilized as the references. According to the experimental data shown in Table 3, **8i** exhibited favorable stability in both HLM (T_1/2_ = 74.9 min) and RLM (T_1/2_ > 145 min), as illustrated by the long elimination half-lives.

**Table 3:** The *in vitro* metabolic stability of compound **8i** in HLM and RLM.

**Compd**.	**HLM**				**RLM**			
	**T_**1/2**_ (min)**	**CL_**int(mic)**_ (μL/min/mg)^**a**^**	**CL_**int(liver)**_ (mL/min/kg)^**b**^**	**Remaining% (T = 60min)**	**T_**1/2**_ (min)**	**CL_**int(mic)**_ (μL/min/mg)^**a**^**	**CL_**int(liver)**_ (mL/min/kg)^**b**^**	**Remaining% (T = 60min)**
**8i**	74.9	18.5	16.6	57.1	>145	< 9.6	< 17.3	72.8
Testosterone	13.7	101.2	91.1	4.8	0.7	2097.5	3775.6	0.0
Diclofenac	9.6	144.3	129.9	1.2	17.4	79.5	143.0	8.7
Propafenone	6.6	211.1	190.0	0.2	2.1	657.9	1184.2	0.3

a *CL_int(mic)_ = 0.693/half life/mg microsome protein per mL*.

b CL_int(liver)_ = ${\text{CL}}_{\text{int}\left(\text{mic}\right)}^{*}$mg microsomal protein/g liver weight^*^g liver weight/kg body weight.

#### Pharmacokinetics

**Figure 4:**
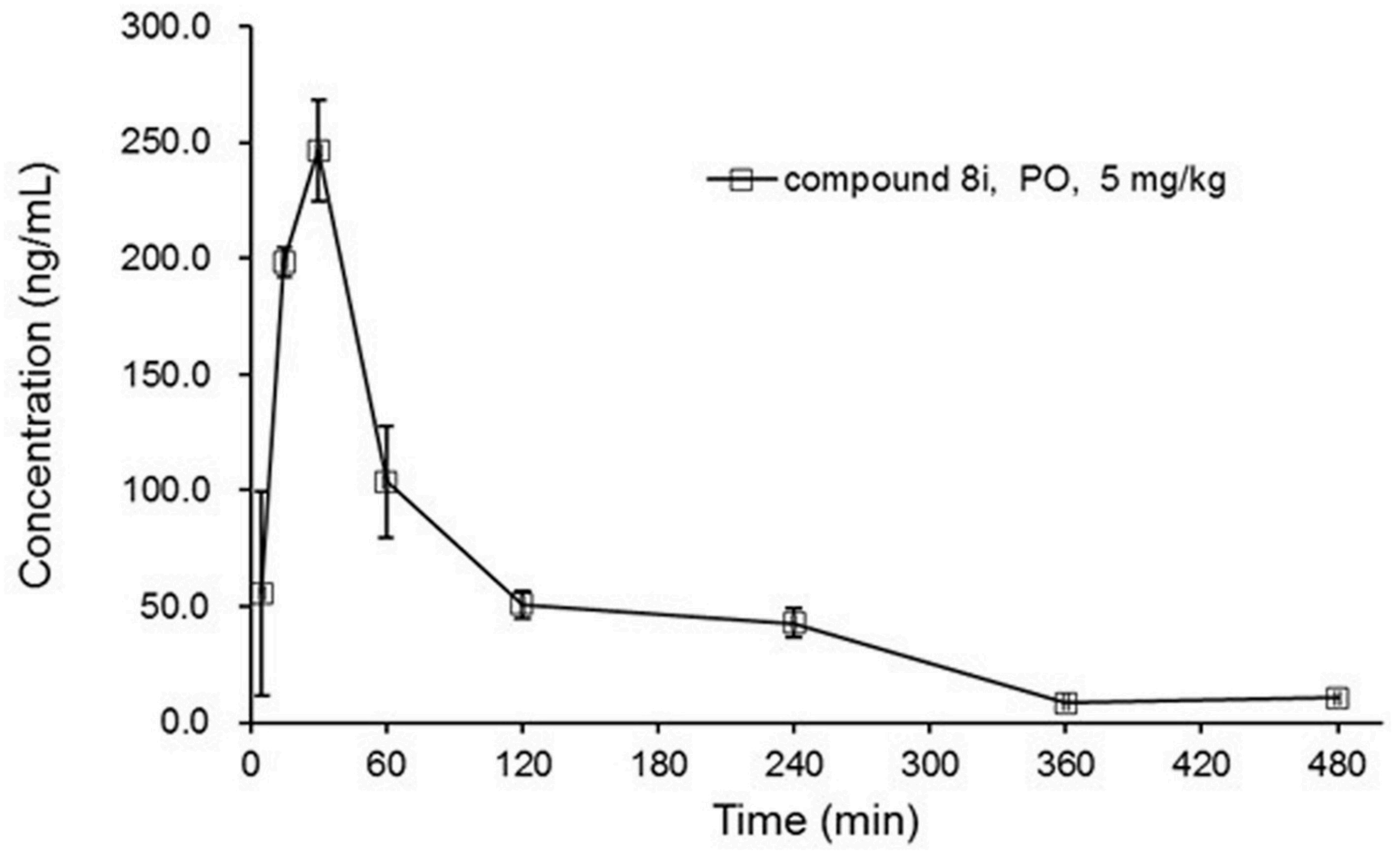
The concentration-time curve of **8i** in SD rats: three animals for PO dose of 5 mg/kg.

By virtue of its favorable *in vitro* potency and metabolic stability, **8i** was further evaluated for its PK profiles in Sprague-Dawley (SD) rats following oral administration at 5 mg/kg (Figure 4). As a result, **8i** showed acceptable plasma exposure (AUC_0−t_ = 379 h•ng/mL; AUC_0−∞_ = 412 h•ng/mL), peak plasma concentration (Cmax = 249 ng/mL), and elimination half-life (t_1/2_ = 1.73 h).

## Conclusion

In summary, a novel series of quinoline derivatives were designed and synthesized as PI3K/mTOR dual inhibitors, *via* introducing acrylamide fragment to the C-4 position for probing residue Gln859. According to the PI3Kα enzymatic assay, all of them were remarkably potent with IC_50_s at sub-nanomolar or low nanomolar level. Consistent with this, they exhibited attractive anti-proliferative efficacy against both PC3 and HCT116 cell lines. To our expectation, **8i**, a representative throughout this series (IC_50_: PI3Kα, 0.50 nM; GI_50_s: PC3, 0.40 μM; HCT116, 0.47 μM), also significantly inhibited other class I PI3Ks and mTOR with all the IC_50_s at sub-nanomolar or low nanomolar level. In the PC3 cell line, **8i** dramatically down-regulated the levels of phos-Akt (Ser473), phos-Akt (Thr308), phos-S6 ribosomal protein (Ser235/236), and phos-4E-BP1 (Thr37/46) at a concentration as low as 5 nM, indicating its considerable capability to ablate PAM signaling. Besides, the concurrent down-regulation of the four biomarkers was beneficial for fulfilling synergism and conquering the negative feedback loop released by mono-inhibition of mTOR. Moreover, **8i** was metabolically stable in both HLM and RLM with long elimination half-life. A further *in vivo* PK study of **8i** in SD rats following oral administration at 5 mg/kg illustrated **8i** possessed acceptable oral exposure, peak plasma concentration, and elimination half-life. According to the above results, it can be concluded that introduction of 4-acrylamide substructure at the C-4 position of the quinoline core was a feasible way to attain potent PI3K/mTOR dual inhibitors with structural novelty. Besides, **8i**, with acceptable *in vitro* and *in vivo* biological profiles, merited further investigation and structural optimization.

## Ethics Statement

The study was carried out in accordance with institutional guidelines of the Animal Research Committee at Jiaxing University (log number JXU2015120812). The protocol was approved by the institution.

## Author Contributions

XL designed and supervised this research and performed the molecular simulation. The chemical synthesis and purification were predominantly conducted by XM and LS. XL and XM were involved in the data analysis and manuscript writing. JZ and GL critically evaluated the scientific work. SZ and BD participated in the biological evaluation of compounds. All authors approved the final manuscript.

### Conflict of Interest Statement

The authors declare that the research was conducted in the absence of any commercial or financial relationships that could be construed as a potential conflict of interest. The reviewer QH declared a shared affiliation, though no other collaboration, with several of the authors LS, JZ to the handling Editor.

## References

[1] Beaufils F. Cmiljanovic N. Cmiljanovic V. Bohnacker T. Melone A. Marone R.. (2017). 5-(4,6-Dimorpholino-1,3,5-triazin-2-yl)-4-(trifluoromethyl)pyridin-2-amine (PQR309), a Potent, Brain-Penetrant, Orally Bioavailable, Pan-Class I PI3K/mTOR inhibitor as clinical candidate in oncology. J. Med. Chem. 60, 7524–7538. 10.1021/acs.jmedchem.7b00930 28829592 PMC5656176

[2] Elkabets M. Vora S. Juric D. Morse N. Mino-Kenudson M. Muranen. (2013). mTORC1 inhibition is required for sensitivity to PI3K p110alpha inhibitors in PIK3CA-mutant breast cancer. Sci. Transl. Med. 5:196ra99. 10.1126/scitranslmed.3005747 23903756 PMC3935768

[3] Folkes A. J. Ahmadi K. Alderton W. K. Alix S. Baker S. J. Box G. (2008). The identification of 2-(1H-indazol-4-yl)-6-(4-methanesulfonyl-piperazin-1-ylmethyl)-4-morpholin-4-yl-t hieno[3,2-d]pyrimidine (GDC-0941) as a potent, selective, orally bioavailable inhibitor of class I PI3 kinase for the treatment of cancer. J. Med. Chem. 51, 5522–5532. 10.1021/jm800295d 18754654

[4] Furet P. Guagnano V. Fairhurst R. A. Imbach-Weese P. Bruce I. Knapp M.. (2013). Discovery of NVP-BYL719 a potent and selective phosphatidylinositol-3 kinase alpha inhibitor selected for clinical evaluation. Bioorg. Med. Chem. Lett. 23, 3741–3748. 10.1016/j.bmcl.2013.05.007 23726034

[5] Garces A. E. Stocks M. E. (2019). Class 1 PI3K clinical candidates and recent inhibitor design strategies: a medicinal chemistry perspective. J. Med. Chem. 10.1021/acs.jmedchem.8b01492. 30582807

[6] Heffron T. P. Heald R. A. Ndubaku C. Wei B. Augistin M. Do S.. (2016). The Rational Design of Selective Benzoxazepin Inhibitors of the alpha-Isoform of Phosphoinositide 3-Kinase Culminating in the Identification of (S)-2-((2-(1-Isopropyl-1H-1,2,4-triazol-5-yl)-5,6-dihydrobenzo[f]imidazo[1,2-d][1,4]oxazepin-9-yl)oxy)propanamide (GDC-0326). J. Med. Chem. 59, 985–1002. 10.1021/acs.jmedchem.5b01483 26741947

[7] Lalonde M. P. McGowan M. A. Rajapaksa N. S. Jacobsen E. N. (2013). Enantioselective formal aza-Diels-Alder reactions of enones with cyclic imines catalyzed by primary aminothioureas. J. Am. Chem. Soc. 135, 1891–1894. 10.1021/ja310718f 23321009 PMC3566267

[8] Liu Q. Chang J. W. Wang J. Kang S. A. Thoreen C. C. Markhard A.. (2010). Discovery of 1-(4-(4-propionylpiperazin-1-yl)-3-(trifluoromethyl)phenyl)-9-(quinolin-3-yl)benzo[h][1,6]naphthyridin-2(1H)-one as a highly potent, selective mammalian target of rapamycin (mTOR) inhibitor for the treatment of cancer. J. Med. Chem. 53, 7146–7155. 10.1021/jm101144f 20860370 PMC3893826

[9] Liu Q. Wang J. Kang S. A. Thoreen C. C. Hur W. Ahmed T.. (2011). Discovery of 9-(6-aminopyridin-3-yl)-1-(3-(trifluoromethyl)phenyl)benzo[h][1,6]naphthyridin-2(1H)-one (Torin2) as a potent, selective, and orally available mammalian target of rapamycin (mTOR) inhibitor for treatment of cancer. J. Med. Chem. 54, 1473–1480. 10.1021/jm101520v 21322566 PMC3090687

[10] Lv X. Ying H. Ma X. Qiu N. Wu P. B. (2015). Design, synthesis and biological evaluation of novel 4-alkynyl-quinoline derivatives as PI3K/mTOR dual inhibitors. Eur. J. Med. Chem. 99, 36–50. 10.1016/j.ejmech.2015.05.025 26046312

[11] Ma X. Hu Y. (2013). Targeting PI3K/Akt/mTOR cascade: the medicinal potential, updated research highlights and challenges ahead. Curr. Med. Chem. 20, 2991–3010. 10.2174/09298673113209990124 23651303

[12] Ma X. Lv X. Qiu N. Yang B. He Q. Hu Y. (2015). Discovery of novel quinoline-based mTOR inhibitors via introducing intra-molecular hydrogen bonding scaffold (iMHBS): The design, synthesis and biological evaluation. Bioorg. Med. Chem. 23, 7585–7596. 10.1016/j.bmc.2015.11.003 26596710

[13] Ma X. Lv X. Zhang J. (2018). Exploiting polypharmacology for improving therapeutic outcome of kinase inhibitors (KIs): an update of recent medicinal chemistry efforts. Eur. J. Med. Chem. 143, 449–463. 10.1016/j.ejmech.2017.11.049 29202407

[14] Ndubaku C. O. Heffron T. P. Staben S. T. Baumgardner M. Blaquiere N. Bradley E. (2013). Discovery of 2-{3-[2-(1-isopropyl-3-methyl-1H-1,2-4-triazol-5-yl)-5,6-dihydrobenzo[f]imidazo[1,2-d][1,4]oxazepin-9-yl]-1H-pyrazol-1-yl}-2-methylpropanamide (GDC-0032): a beta-sparing phosphoinositide 3-kinase inhibitor with high unbound exposure and robust *in vivo* antitumor activity. J. Med. Chem. 56, 4597–4610. 10.1021/jm4003632 23662903

[15] Perez-Melero C. Maya A. B. Rey B. del Pelaez R. Caballero E. Medarde M. (2004). A new family of quinoline and quinoxaline analogues of combretastatins. Bioorg. Med. Chem. Lett. 14, 3771–3774. 10.1016/j.bmcl.2004.04.098 15203159

[16] Phanumartwiwath A. Hornsby T. W. Jamalis J. Bailey C. D. Willis C. L. (2013). Silyl migrations in D-xylose derivatives: total synthesis of a marine quinoline alkaloid. Org. Lett. 15, 5734–5737. 10.1021/ol402760p 24229077

[17] Pike K. G. Morris J. Ruston L. Pass S. L. Greenwood R. Williams E. J.. (2015). Discovery of AZD3147: a potent, selective dual inhibitor of mTORC1 and mTORC2. J. Med. Chem. 58, 2326–2349. 10.1021/jm501778s 25643210

[18] Stauffer F. Maira S. M. Furet P. Garcia-Echeverria C. (2008). Imidazo[4,5-c]quinolines as inhibitors of the PI3K/PKB-pathway. Bioorg. Med. Chem. Lett. 18, 1027–1030. 10.1016/j.bmcl.2007.12.018 18248814

[19] Yadav R. R. Guru S. K. Joshi P. Mahajan G. Mintoo M. J. Kumar V.. (2016). 6-aryl substituted 4-(4-cyanomethyl) phenylamino quinazolines as a new class of isoform-selective PI3K-alpha inhibitors. Eur. J. Med. Chem. 122, 731–743. 10.1016/j.ejmech.2016.07.006 27479483

[20] Yang H. Rudge D. G. Koos J. D. Vaidialingam B. Yang H. J. Pavletich N. P. (2013). mTOR kinase structure, mechanism and regulation. Nature 497, 217–223. 10.1038/nature12122 23636326 PMC4512754

[21] Zhang J. Lv X. Ma X. Hu Y. (2017a). Discovery of a series of *N*-(5-(quinolin-6-yl)pyridin-3-yl)benzenesulfonamides as PI3K/mTOR dual inhibitors. Eur. J. Med. Chem. 127, 509–520. 10.1016/j.ejmech.2017.01.016 28109945

[22] Zhang J. Ma X. Lv X. Li M. Zhao Y. Liu G. (2017b). Identification of 3-amidoquinoline derivatives as PI3K/mTOR dual inhibitors with potential for cancer therapy. RSC Adv. 7, 2342–2350. 10.1039/C6RA26971K

