# Supplementary material for: Novel 4-Acrylamido-Quinoline Derivatives as Potent PI3K/mTOR Dual Inhibitors: The Design, Synthesis, and in vitro and in vivo Biological Evaluation
Source: Front Chem. 2019 Apr 24;7:236. doi: 10.3389/fchem.2019.00236 (PMC6491818; doi:10.3389/fchem.2019.00236)
Supplement: Supplementary file 1 [file Table_1.DOCX]

***Supplementary Material***

**Novel 4-****acrylamido-quinoline derivatives as potent PI3K/mTOR dual inhibitors: the design, synthesis, and *in vitro* and *in vivo* biological evaluation**

Xiaodong Ma ^1, 2 #^, Li Shen ^3 #^, Jiankang Zhang ^4^, Guoqiang Liu ^5^, Shuyu Zhan ^5^, Baoyue Ding ^5^ and Xiaoqing Lv ^5^ *

^1^School of Pharmacy, Anhui University of Chinese Medicine, Hefei, China

^2^Department of Medicinal Chemistry, Anhui Academy of Chinese Medicine, Hefei, China

^3^Ocean College, Zhejiang University, Zhoushan, China

^4^Zhejiang University City College, Hangzhou, China

^5^College of Medicine, Jiaxing University, Jiaxing, China

**Copies of NMR, HRMS spectra of target compounds**

**^1^H,^13^C NMR and HRMS spectra of compound 8a**

**^1^H,^13^C NMR and HRMS spectra of compound 8b**

**^1^H and HRMS spectra of compound 8c**

**^^**

**^1^H,^13^C NMR and HRMS spectra of compound 8d**

**^1^H,^13^C NMR and HRMS spectra of compound 8e**

**^1^H,^13^C NMR and HRMS spectra of compound 8f**

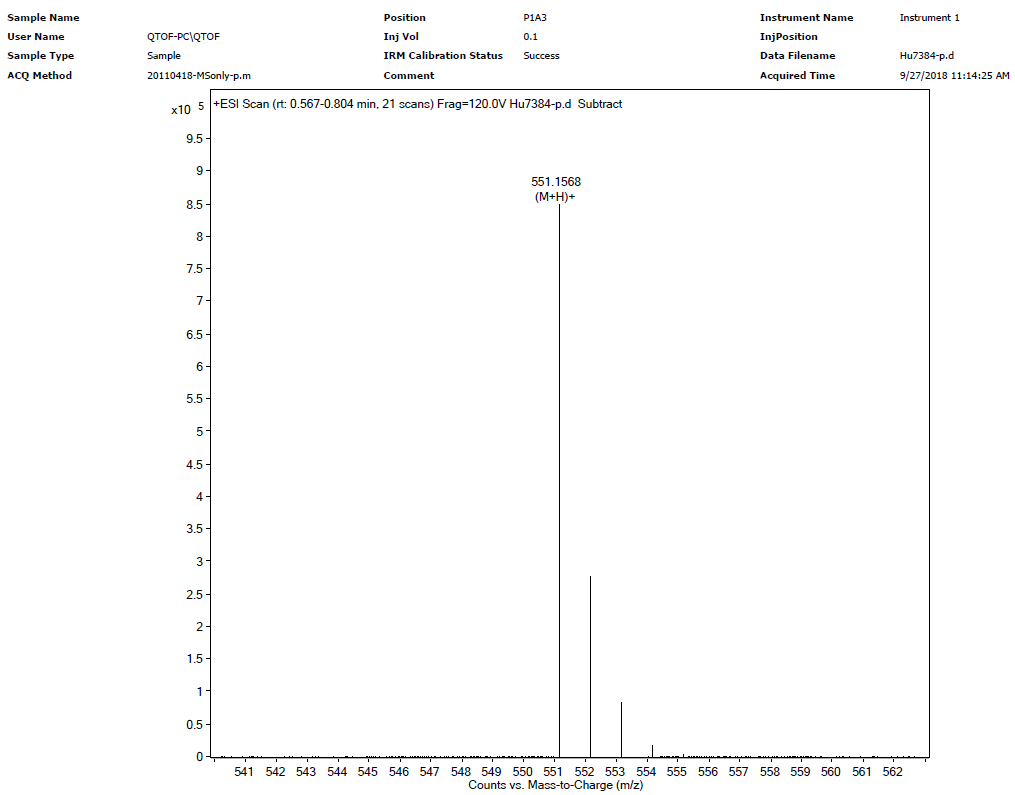


**^1^H,^13^C NMR and HRMS spectra of compound 8g**

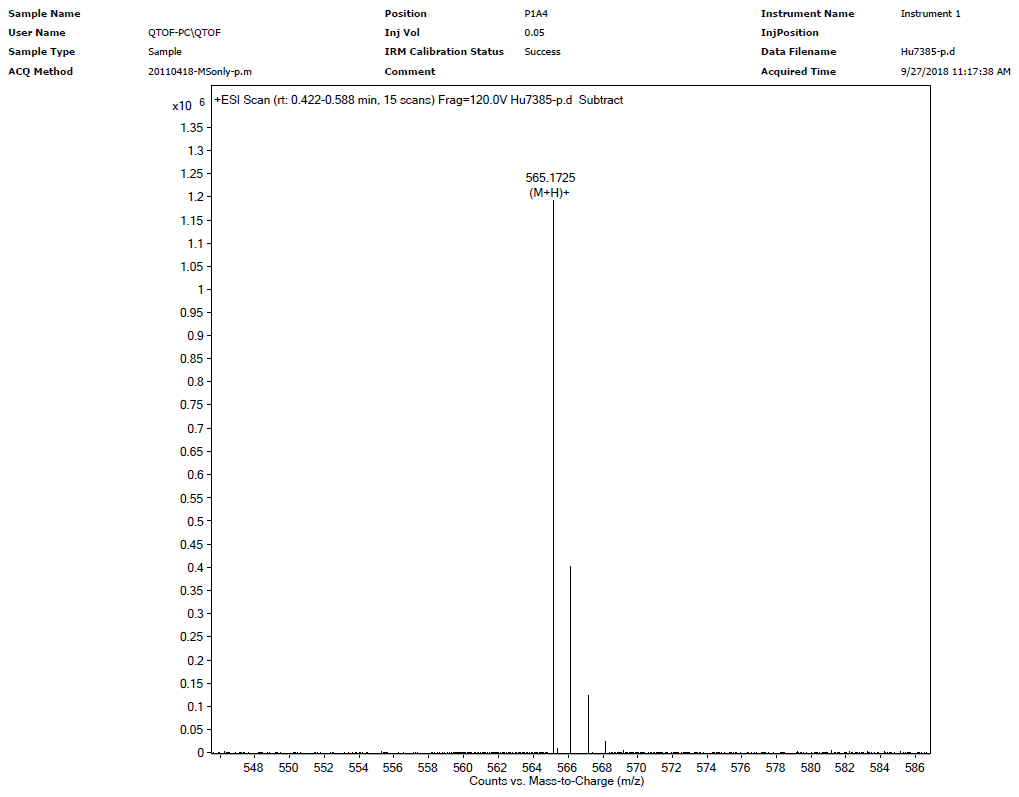


**^1^H,^13^C NMR and HRMS spectra of compound 8h**

**^1^H and HRMS spectra of compound 8i**

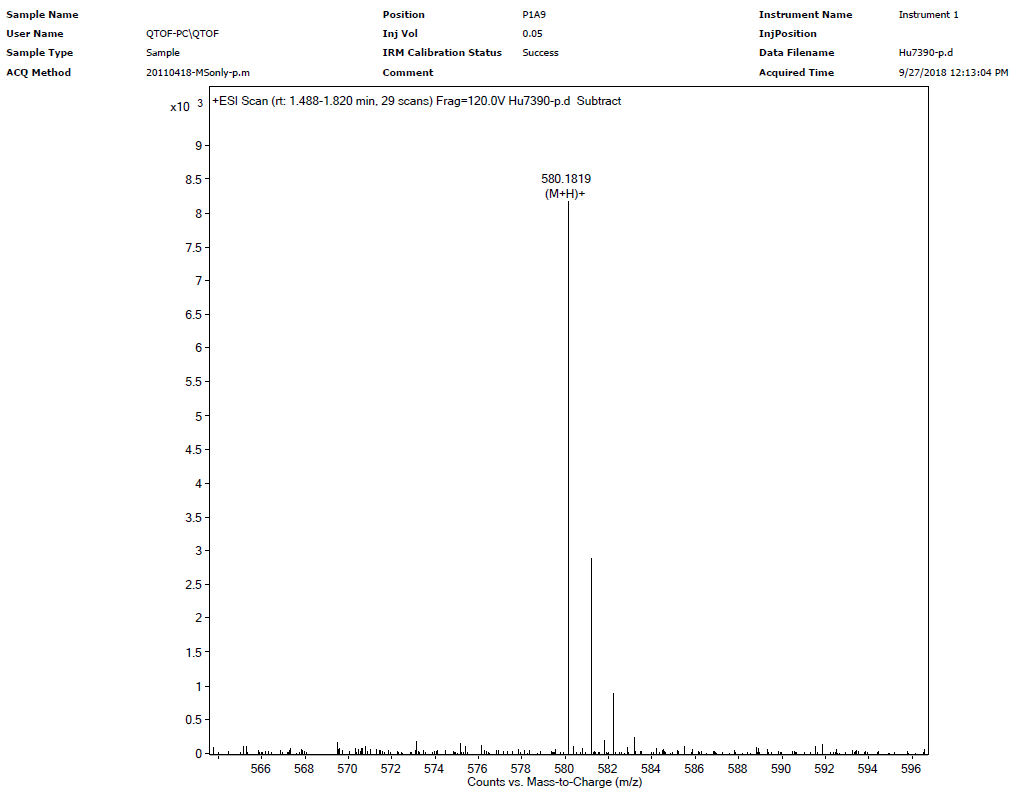


**^1^H,^13^C NMR and HRMS spectra of compound 8j**

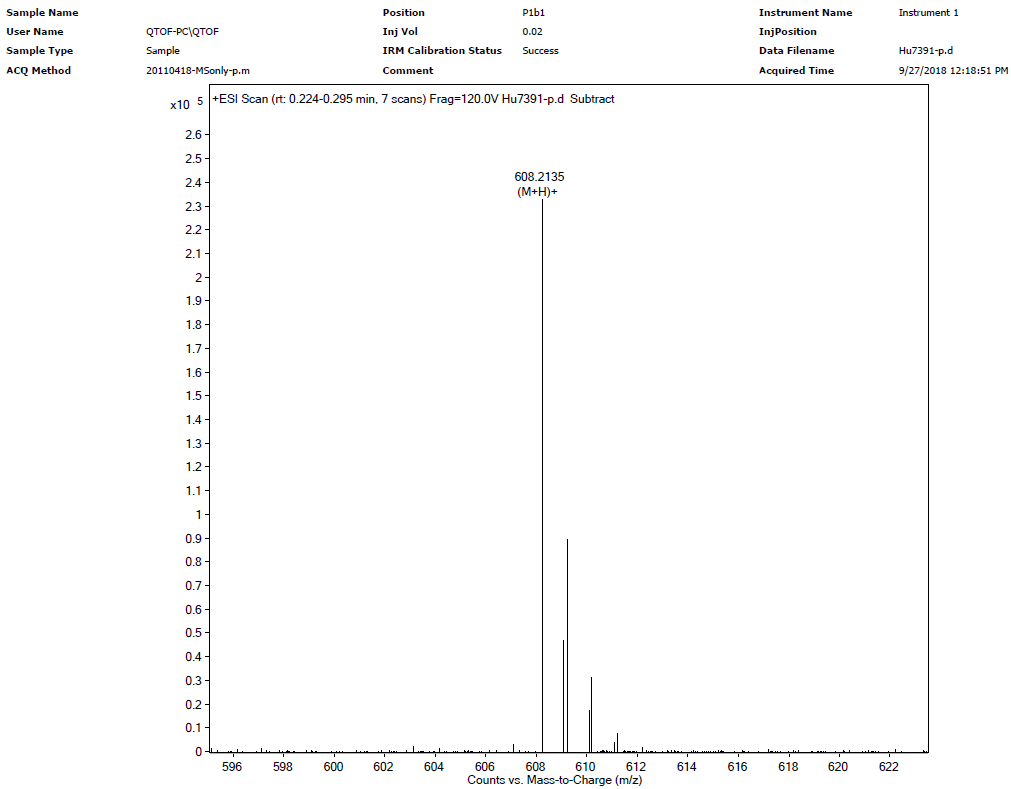


**^1^H,^13^C NMR and HRMS spectra of compound 8k**

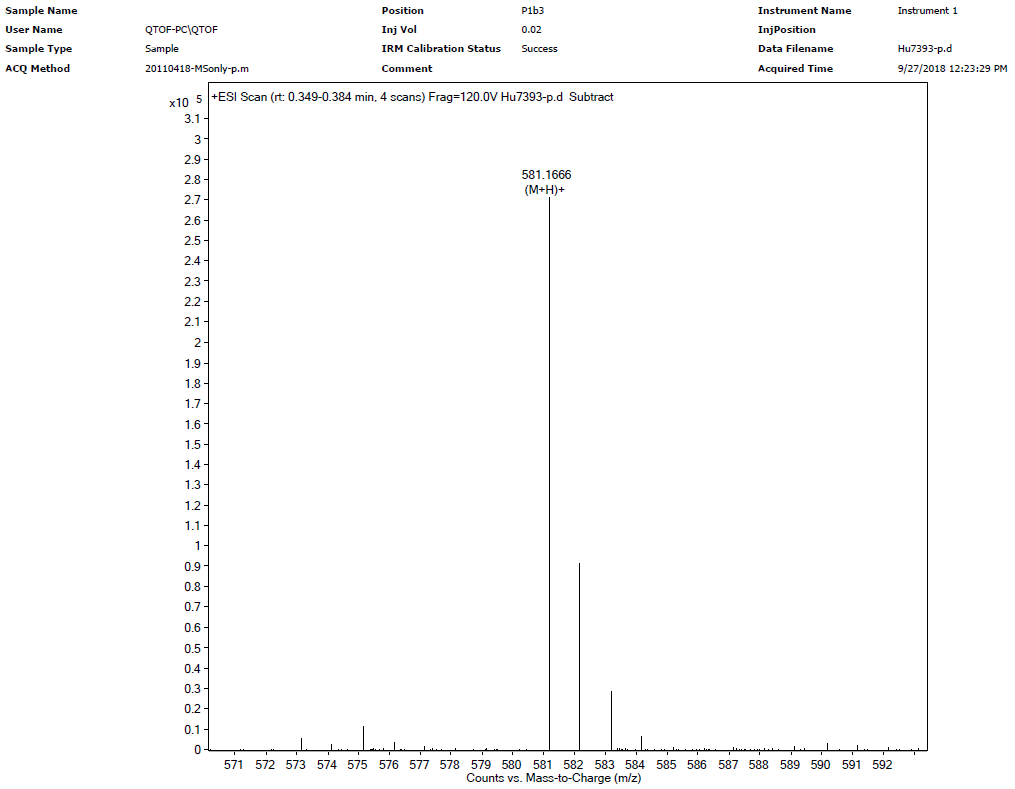


**^1^H,^13^C NMR and HRMS spectra of compound 8l**

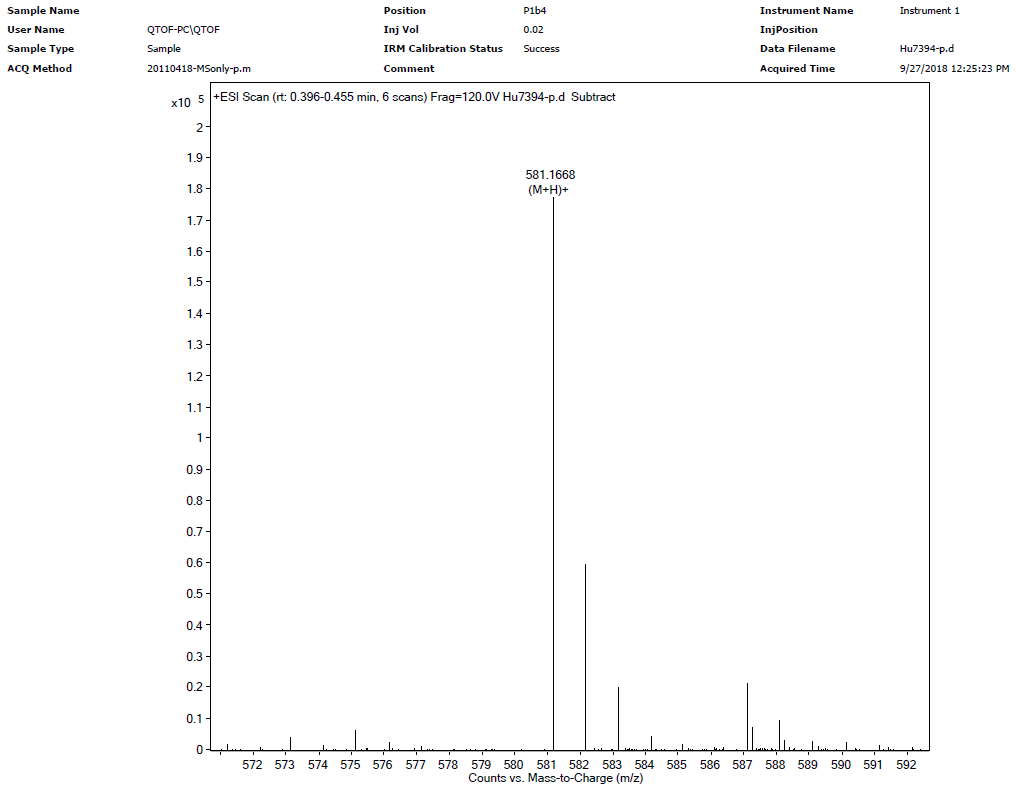


**^1^H,^13^C NMR and HRMS spectra of compound 8m**

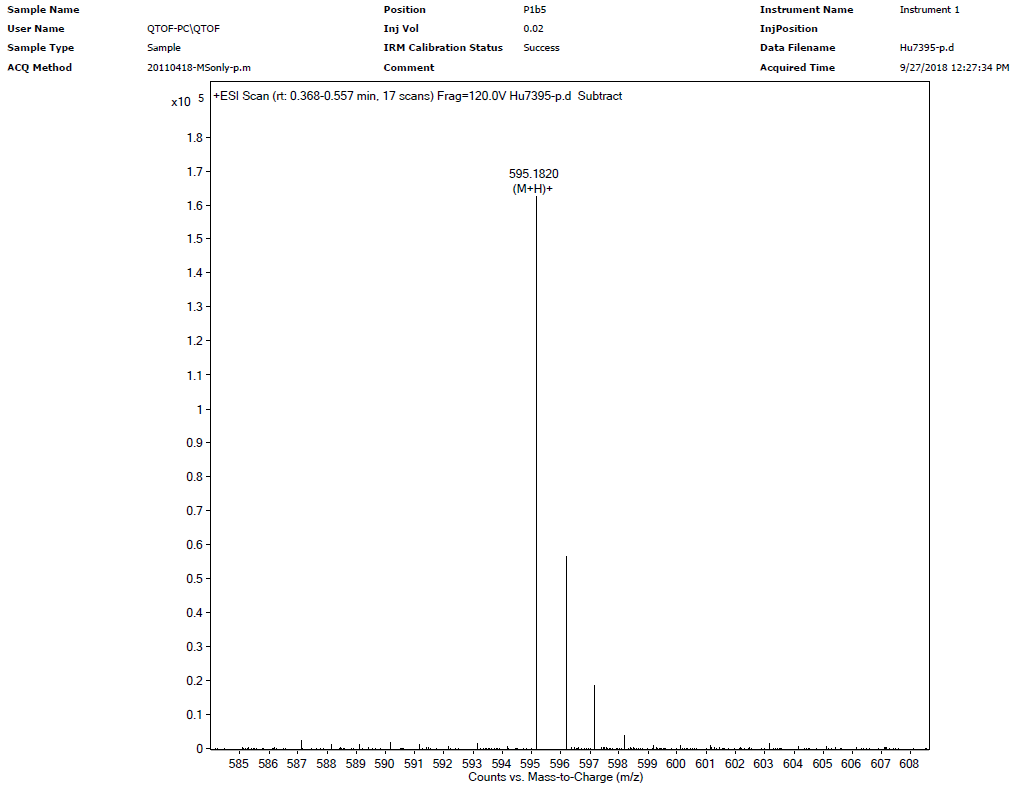


**^1^H and HRMS spectra of compound 8n**

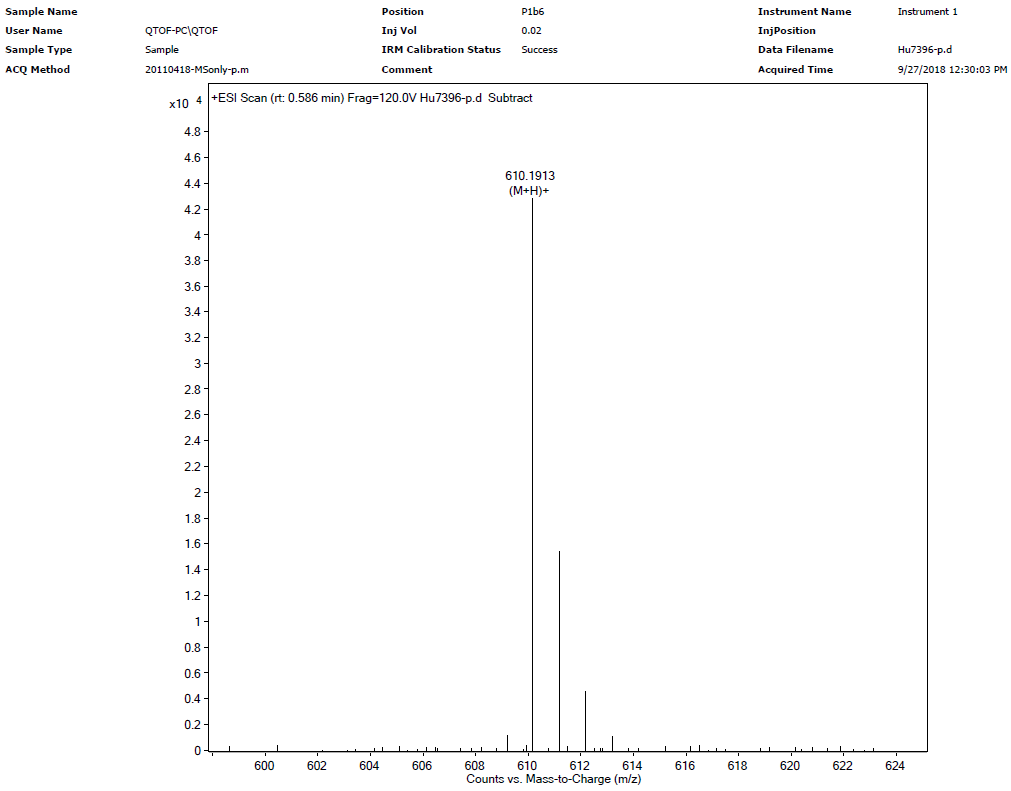


**^1^H,^13^C NMR and HRMS spectra of compound 8o**

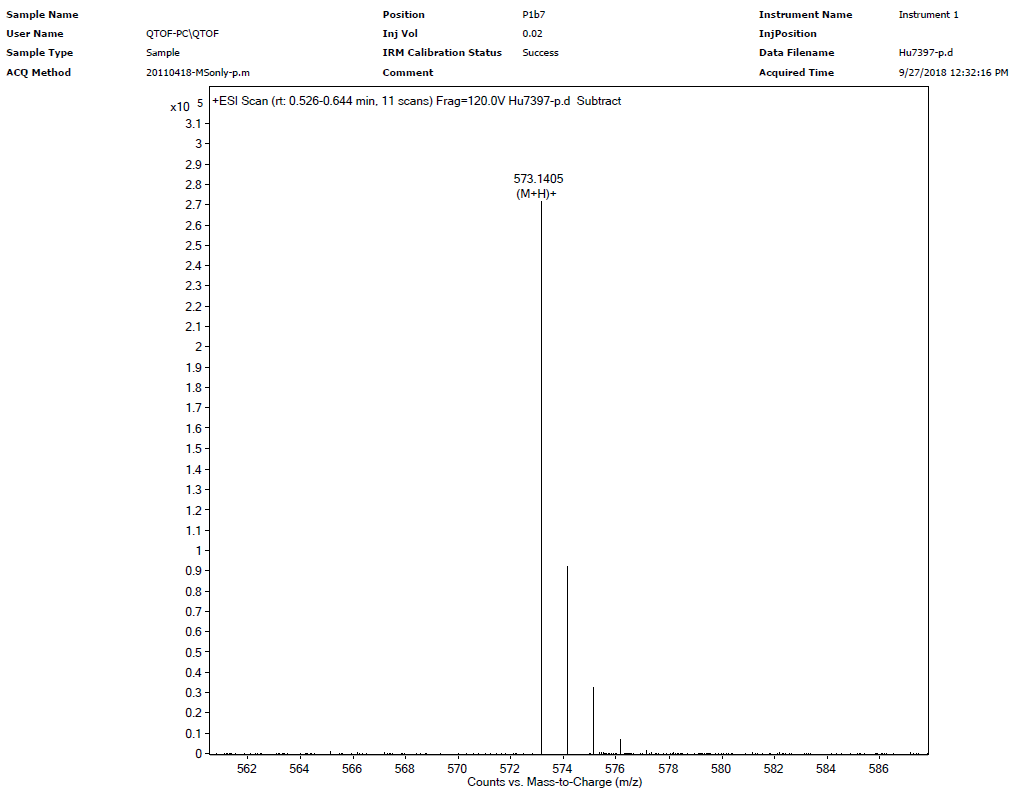


**^1^H and ^13^C NMR spectra of compound 10**
